# Protein Catalysis Through Structural Dynamics: A Comprehensive Analysis of Energy Conversion in Enzymatic Systems and Its Computational Limitations

**DOI:** 10.3390/ph18070951

**Published:** 2025-06-24

**Authors:** Sarfaraz K. Niazi

**Affiliations:** College of Pharmacy, University of Illinois, Chicago, IL 60612, USA; sniazi3@uic.edu; Tel.: +1-312-297-0000

**Keywords:** protein structure, catalysis, enzymatic systems, computational limitations, energy conversion

## Abstract

This review investigates the novel idea that proteins catalyze chemical reactions through conformational changes driven by energy derived from their collisions with water molecules. Recent studies have suggested that proteins in solution undergo constant deformation due to collisions with water molecules, generating potential energy that can be harnessed for catalytic functions. We detail the existing evidence supporting this idea, including how structures in proteins such as α-helices and β-sheets facilitate energy conversion, how conformational changes can affect the ways in which substrates attach, and how reactions occur. Combining information from computer-based methods—such as molecular dynamics simulations and machine learning models (e.g., AlphaFold)—we suggest a more complete model for understanding how proteins function beyond simply looking at their fixed shapes. This emerging view has implications for drug design, enzyme engineering, and our fundamental understanding of biological catalysis.

## 1. Introduction

### 1.1. The Limitations of Static Protein Structure Models

Proteins have traditionally been studied as static entities, with their three-dimensional structures considered the primary determinant of their function. This “structure determines function” paradigm has dominated biochemistry for decades, driving significant advances in structural biology techniques such as X-ray crystallography and cryo-electron microscopy. The Protein Data Bank now contains over 200,000 protein structures, which offer priceless knowledge about the architecture and organization of proteins [1]. However, this static view creates significant limitations when attempting to understand the true nature of protein functions, particularly in the context of enzymatic catalysis.

Crystallographic structures represent proteins in their most stable form, usually under unusual conditions such as very low temperatures, lack of water, or being trapped in a crystal. These structures offer a snapshot of a single conformational state, failing to capture the dynamic reality of proteins in solution. As Henzler-Wildman and Kern [2] noted, proteins exist as “dynamic ensembles” rather than rigid structures, adopting multiple conformations that may be critical for their biological function. The limitations of static models become particularly apparent when examining catalytic mechanisms, in which transient states and conformational changes often play essential roles.

Recent technological advances have begun to illuminate the inadequacy of static models. Time-resolved crystallography, solution NMR, and molecular dynamics simulations have shown that proteins often change shape while functioning as catalysts [3]. These studies suggest that the dynamic properties of proteins are not merely incidental to their function but are fundamental to their catalytic mechanisms. Despite these advances, there remains a significant gap in our understanding of how protein dynamics contribute to catalytic efficiency and specificity.

**Figure 1:**
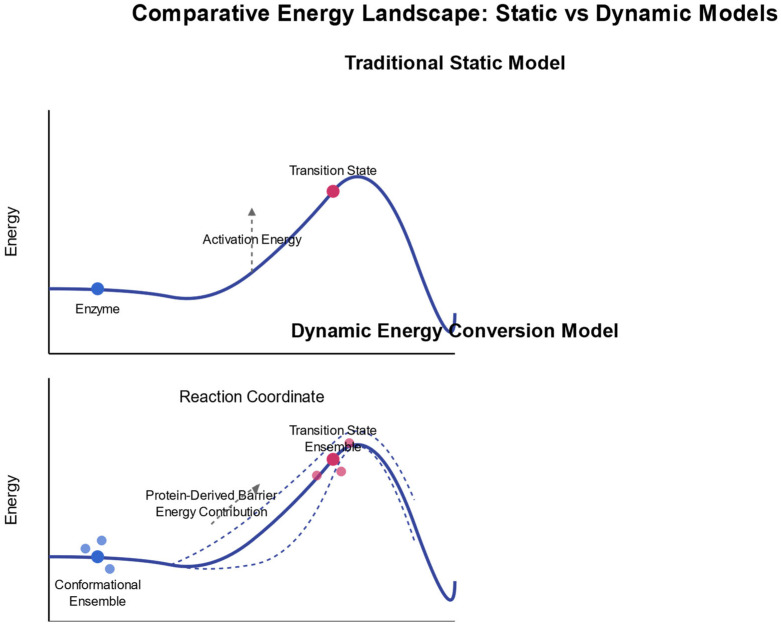
Comprehensive framework for understanding protein dynamics as an energy conversion system.

Figure 1 illustrates the key distinctions between static and dynamic energy landscape models in enzymatic reactions. The comparative diagram highlights three fundamental differences: (1) energy sources—the static model relies solely on binding energy, whereas the dynamic model incorporates both binding energy and protein conformational energy derived from Brownian motion; (2) reaction coordinates—the static model follows a single, well-defined pathway with fixed transition state geometry and assumes rigid conformations, while the dynamic model encompasses multiple pathways through conformational space influenced by thermal fluctuations, considering transition state ensembles rather than discrete states; and (3) experimental predictions—the static model suggests that structure determines reaction rates and that changes far from the active site have minimal effects, whereas the dynamic model demonstrates that dynamics significantly impact reaction rates, allowing mutations distant from the active site to substantially alter enzymatic activity. The upper portion of the figure depicts the traditional static model, showing a simple energy curve with a single activation barrier, while the lower portion illustrates the dynamic energy conversion model, presenting multiple potential pathways and conformational ensembles at both reactant and transition states.

### 1.2. The Historical Context of Protein Dynamics in Catalysis

The recognition that protein dynamics play a crucial role in catalysis has evolved gradually over several decades. Early signs included the indication that, in theoretical models of enzyme catalysis, proteins need to change shape to bind substrates, carry out chemical reactions, and release products. Koshland’s “induced fit” model [4] represented one of the first formal acknowledgments that proteins could adapt their structure upon substrate binding, challenging the rigid “lock and key” model proposed by Fischer in 1894.

By the 1970s, experimental evidence supporting the importance of protein dynamics began accumulating. Relaxation studies, hydrogen–deuterium exchange experiments, and fluorescence spectroscopy analyses revealed that proteins move differently, from fast bond vibrations to slower movements in larger parts. Frauenfelder and his team [5] showed that proteins are not just present in one shape but are constantly changing between different forms due to thermal energy.

The 1990s and early 2000s saw significant advances in both experimental and computational techniques for studying protein dynamics. Solution NMR methods, such as that developed by Wüthrich and others, enable detailed characterization of the motion of proteins in solution [6]. At the same time, better computer technology enabled more accurate molecular dynamics simulations, helping researchers to visualize protein movements in detail over extended periods.

A pivotal shift occurred with studies demonstrating direct correlations between protein dynamics and catalytic activity. Significant research on dihydrofolate reductase by Benkovic and Hammes-Schiffer [3] demonstrated that movements in the protein far from the active site could affect the reaction rate via a system of connected movements. This work and similar studies established that dynamic effects are not merely correlated with catalysis but could be causally linked to catalytic function.

### 1.3. Emerging Paradigm: Proteins as Dynamic Energy Converters

The dynamic energy conversion model proposes three fundamental principles. First, proteins in solution constantly take in kinetic energy through collisions with the fast-moving water molecules around them via Brownian motion, which occurs at a very high rate (10^9^–10^12^ times per second) and provides a steady supply of heat energy to the protein structure. Second, this kinetic energy is turned into potential energy that is held within the protein structure, especially in secondary structures such as α-helices and β-sheets, which are good at storing energy due to their regular shapes and hydrogen bonds that allow for different modes of bending and stretching. Third, the stored potential energy is directed to catalytic sites, where it reduces the energy needed for reactions (usually between 20 and 40 kJ/mol for enzyme reactions) and aids in chemical modifications; for example, this energy can weaken bonds in the substrate, support transition states, or enable the conformational changes required for catalysis. This model conceptualizes proteins not as passive scaffolds for positioning reactive groups but instead as active mechanical systems that directly contribute energy to catalytic reactions.

Building on this core hypothesis, an emerging paradigm proposes a more mechanistic understanding of how protein dynamics contribute to catalysis. This new model, suggested by Cao and Ding [7] and backed by other researchers, poses proteins as active energy converters that use the movement of water molecules to create energy for chemical reactions.

Water molecules and other particles continuously bombard proteins in solution, causing structural deformations that generate internal stress. This stress—mainly when focused on structures such as α-helices and β-sheets—builds up potential energy that can be used to break chemical bonds in substrate molecules. Unlike the static view, where proteins are considered to provide a passive scaffold for bringing reactive groups into proximity, this dynamic model suggests that amino acids actively contribute energy to catalytic reactions through their structural dynamics.

This paradigm offers several advantages for understanding enzyme catalysis. First, it provides a physical mechanism through which enzymes lower activation energy barriers beyond simply positioning substrates or stabilizing transition states. Second, it explains the temperature dependence of enzyme activity as a function of increased molecular motion and collision frequency. Third, it accounts for the role of protein flexibility in catalysis; particularly the observation that excessively rigid proteins often display reduced catalytic activity.

Recent experimental and computational studies have begun to provide evidence supporting this energy conversion model. Zhou et al. [8] demonstrated that protein conformational dynamics can directly contribute to the energy needed for chemical transformations. Similarly, research on adenylate kinase by Henzler-Wildman et al. [9] showed that the changes in an enzyme’s shape are closely linked to its process of speeding up reactions, with specific movements affecting different parts of the reaction.

### 1.4. Scope and Objectives of This Review

This review aims to comprehensively examine the emerging paradigm of proteins as dynamic energy converters in enzymatic catalysis. We analyze the theoretical foundations of this model, evaluate the experimental and computational evidence supporting it, and explore its implications for our understanding of the functions and applications of protein in biotechnology and medicine.

Specifically, we establish a theoretical framework for understanding how proteins harness and convert energy derived from Brownian motion into catalytic potential. We examine the roles of secondary structures—particularly α-helices and β-sheets—as key elements involved in energy absorption and conversion. We look at how changes in protein shape affect the structure of the part that helps with chemical reactions, and how these changes impact how well the protein can bind to substances and carry out chemical modifications.

We further review experimental methodologies for studying protein dynamics at various timescales and evaluate the evidence they provide for dynamic catalytic models. We discuss how computer methods—such as molecular dynamics simulations and machine learning models like AlphaFold—help us to predict and study how proteins change during catalytic reactions.

The implications of this dynamic model for drug design, enzyme engineering, and our understanding of protein evolution are then explored. Finally, we identify challenges and future directions for research in this field, highlighting key questions that remain to be addressed.

**Figure 2:**
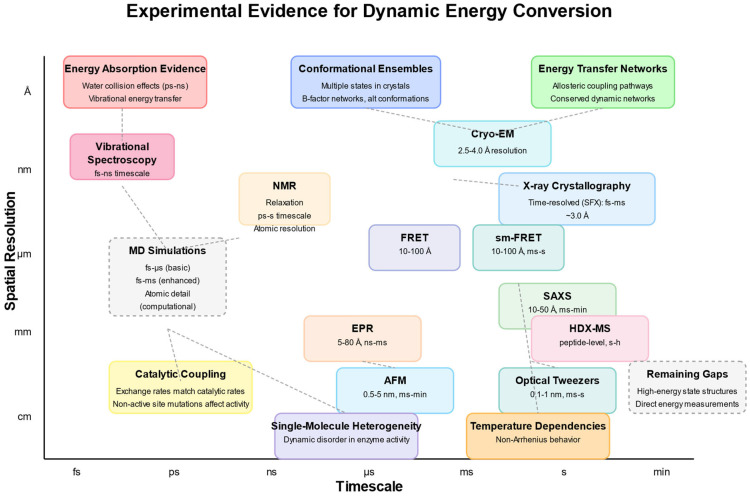
Experimental evidence for dynamic energy conversion activity of proteins.

Through combining knowledge from the domains of structural biology, biophysics, computational biology, and biochemistry, we aim to provide a complete overview of how protein movement plays a role in catalysis and suggest a unified way to view proteins as active energy-converting machines instead of simply as fixed structures. In this line, Figure 2 maps the diverse experimental techniques providing evidence for the dynamic conversion of energy by proteins across spatial (Å to cm) and temporal (fs to min) scales. Three categories of evidence are highlighted: energy absorption evidence (including water collision effects and vibrational energy transfer), conformational ensembles (showing multiple states in crystals and B-factor networks), and energy transfer networks (demonstrating allosteric coupling pathways and conserved dynamic networks). Experimental approaches span from ultrafast techniques such as vibrational spectroscopy (fs-ns timescale) to those carried out on a longer timescale such as HDX-MS (s-h) and optical tweezers (ms-s), while the spatial resolution ranges from atomic-level detail in MD simulations and NMR to larger-scale measurements obtained via AFM (0.5–5 nm). Functional evidence supporting dynamic energy conversion includes catalytic coupling (where exchange rates match catalytic rates and nonactive site mutations affect activity), single-molecule heterogeneity (revealing dynamic disorder in enzyme activity), and temperature dependencies (showing non-Arrhenius behavior). The diagram also identifies remaining gaps in the field, particularly those relating to characterizing high-energy state structures and directly measuring energy conversion. Together, these complementary approaches validate proteins as dynamic energy converters functioning across multiple spatiotemporal scales rather than static structural templates (Figure 2).

#### Relationship to Traditional Catalytic Models

The dynamic energy conversion model does not dismiss traditional theories of enzyme catalysis; instead, it adds to them by taking into consideration the roles of the movement and energy conversion functions of proteins. Understanding how this model relates to established catalytic theories is essential for its proper contextualization.

### 1.5. Complementary Aspects of Transition State Theory

Transition state theory (TST) remains the fundamental framework for understanding chemical reactions, including enzymatic catalysis. The dynamic energy conversion model works well with TST, explaining how enzymes help to reduce the energy needed for responses to reach transition states. Traditional TST looks at how enzymes help to stabilize transition states through good interactions. At the same time, the dynamic model suggests that proteins can also provide energy to assist reactants in reaching those transition states. While TST primarily considers energetic contributions from binding interactions, the dynamic model includes mechanical energy derived from the protein’s motion. The “catalytic strain” concept in traditional models aligns well with the dynamic model’s emphasis on stored potential energy, providing a physical basis for how strain is generated and utilized. The dynamic energy conversion model diverges from traditional static models in several ways; for example, static models typically view proteins as relatively rigid scaffolds that position reactants and catalytic groups, whereas the dynamic model treats proteins as mechanical systems that actively convert and transmit energy. Traditional methods usually look at the types of amino acids present in active sites, while the dynamic model further highlights how the overall shape and movement of the protein are crucial in determining how well it works as a catalyst. In static models, it is usually considered that the speeding up of reactions mainly occurs because the transition state is stabilized, while the dynamic model argues that adding mechanical energy to destabilize the starting state is also very important. Additionally, while traditional models consider the contributions of heat energy to catalysis, the dynamic model also considers the effects of protein movements.

### 1.6. Experimental Tests to Distinguish Between Models

Several experimental approaches could help to differentiate between traditional static and dynamic energy conversion models. The isotope-effects-beyond-active-sites method involves assessing the effects of heavy isotopes (e.g., deuterium or carbon-13) far from the active site, which, if the dynamic model is right, should alter the speed of reactions by changing how molecules vibrate and transfer energy. Dependence analysis is significant in this context, as the dynamic model predicts specific non-Arrhenius behaviors in temperature-dependence studies, with distinct effects on rate constants compared to static models. Effects-based studies can help to distinguish between volume-based effects (which are important in traditional models) and compressibility effects (which are critical in the dynamic model). Effects are relevant because changes in solvent viscosity should impact the energy transferred through water collisions, affecting catalytic rates in ways not predicted by static models. Interesting tests—such as creating enzymes with altered mechanical properties but preserved active site geometry—could enable direct testing of the dynamic model’s predictions. These approaches collectively offer ways to validate the dynamic energy model’s distinctive features experimentally. By examining these complementary and divergent aspects, we can develop a more sophisticated understanding of enzyme catalysis that incorporates the well-established principles of transition state theory and emerging insights from the dynamic energy conversion model.

## 2. Theoretical Framework for Protein Dynamics and Energy Conversion

### 2.1. Brownian Motion and Its Impact on Protein Structure

Brownian motion—namely, the random movement of particles suspended in a fluid resulting from collision with fast-moving molecules—was first described by Robert Brown in 1827 and later explained by Albert Einstein in 1905. This phenomenon plays a crucial role in molecular interactions in biological systems, particularly for proteins in aqueous environments. While traditionally viewed as a source of noise or randomness, emerging evidence suggests that Brownian motion may serve as a fundamental energy source for protein functions.

Proteins in solution are continuously bombarded by moving water molecules which carry thermal energy. Di Rienzo et al. [10] described these collisions at microscopic timescales, with proteins experiencing thousands of impacts per microsecond. Each collision imparts a small amount of momentum to the protein, causing localized deformations in its structure. While individual collisions produce minimal effects, their cumulative impact generates significant conformational changes that propagate throughout the protein’s structure.

The impacts of Brownian motion on proteins can be conceptualized at multiple scales. At the atomic level, collisions with water molecules cause fluctuations in bond lengths, angles, and torsional rotations. These microscopic perturbations translate to larger-scale movements at the level of secondary and tertiary structures. As mentioned by Henzler-Wildman and Kern [2], protein movements happen at different speeds, ranging from high-speed bond vibrations that last femtoseconds to slower domain movements that take milliseconds, where each type of movement is affected by random collisions.

Importantly, proteins are not merely passive recipients of these collisions. Their complex three-dimensional structures, evolved over billions of years, effectively channel this random energy into specific conformational changes. As mentioned by Müller et al. [11], some parts of the protein may serve as “energy pathways,” guiding the energy from water collisions to certain areas of the protein where it can facilitate catalytic functions.

#### Quantification of Molecular Potential Energy Generation

We can quantify the conversion of kinetic energy from water molecules into potential energy within protein structures using principles of classical mechanics and statistical thermodynamics. Following the approach outlined by Cao and Ding [7], we can express this energy conversion process mathematically.

When a protein is impacted by water molecules (or other particles) with a collective momentum of MV, part of this momentum is converted into the protein’s motion (MpVp). At the same time, the remainder is transferred back to the water molecules (MV), which rebound after collision. The potential energy (Ep) gained by the protein through this process can be described as follows:

$$E_{p}=\sum_{k=1}^{n_{1}}\frac{1}{2}{m_{n_{1}}v_{n_{1}}}^{2}-\frac{1}{2}m_{p}v_{p}2-\sum_{k=1}^{n_{2}}\frac{1}{2}{m_{n_{2}}v_{n_{2}}}^{2}$$

This equation represents the difference between the initial kinetic energy of the colliding water molecules and the final kinetic energy of both the protein and the rebounding water molecules. The protein structure stores the remainder as potential energy, primarily through the deformation of covalent and non-covalent bonds.

For catalysis to occur, this potential energy must reach or exceed the energy required to break or form specific chemical bonds in a substrate. The catalytic ability of an enzyme can, therefore, be conceptualized as the frequency with which its total potential energy reaches the threshold necessary for catalysis. This frequency is influenced by factors such as temperature, which affects the velocity and collision frequency of the water molecules, and the solution’s viscosity, which affects the efficiency of momentum transfer.

### 2.2. Energy Transfer Mechanisms in Protein Secondary Structures

The conversion of kinetic energy from water collisions into potential energy is not uniform throughout a protein’s structure. Secondary structures—especially α-helices and β-sheets—are very important in the context of energy storage and transfer due to their consistent shape and how they work together.

α-helices, which are made up of closely packed amino acids linked by hydrogen bonds, can efficiently take in and pass on energy because of their spiral shape. When impacted by water molecules, the helix can undergo various deformations, including compression, extension, bending, and twisting. These changes enable the storage of potential energy in the hydrogen bonds that keep the helix stable and bend the connections in the peptide backbone.

Similarly, β-sheets provide an effective mechanism for energy absorption and transfer. Stabilized by hydrogen bonds between adjacent strands, their extended structure allows for collective movements that distribute energy throughout the sheet. When pushed or pulled, β-sheets can hold energy by stretching or compressing the hydrogen bonds and changing the angle of the peptide backbone.

Super-secondary structures—such as the βαβ motif, which is common in nucleotide-binding domains—may be particularly effective in terms of energy conversion. These complex structural arrangements can channel energy from surface-exposed regions to the protein core, where catalytic sites are often located. As proposed by Miyashita et al. [12], these energy conduits may have evolved specifically to harness Brownian motion for the promotion of catalytic functions.

## 3. Mathematical Models of Protein Deformation Under External Forces

The deformation of protein structures under external forces can be modeled using principles from elasticity theory and molecular mechanics. For small deformations, proteins typically behave as elastic bodies, returning to their original conformation when the deforming force is removed. This elastic behavior can be described using Hooke’s law, which states that the force required to extend or compress a spring is proportional to the distance of extension or compression.

When applied to protein secondary structures, this principle can be expressed as

∑mv = K∆L

where ∑mv is the total momentum transferred to the protein, K is a number that indicates how stiff the structure is, and ∆L denotes how much the structure moves or changes shape. The coefficient K varies depending on the type of secondary structure, its length, and its amino acid composition, with more rigid structures having higher K values.

More advanced models consider how proteins stretch and change shape over time, understanding that this includes both quick and slower, more gradual changes. These models, which are often used in computer simulations, consider the complicated energy changes in proteins and the different speeds at which they change shape.

Recent advances in computational methods have enabled increasingly realistic simulations of protein dynamics under the influence of water collisions. As explained by McGuffee and Elcock [13], molecular dynamics simulations can now closely mimic how proteins move in water, considering the detailed interactions between proteins and their watery surroundings. These simulations offer helpful insights into how the energy derived from water collisions is absorbed, distributed, and utilized within protein structures.

### Mechanistic Framework of Protein Energy Conversion

Proteins convert kinetic energy from water collisions into catalytically sound potential energy, which can be systematized into a comprehensive mechanistic framework. This framework explains how proteins change shape, transfer energy, and perform certain functions in terms of their structures.

Structural elements exhibit characteristic deformation modes which determine their energy absorption and storage capabilities. For example, α-helices demonstrate several deformation modes including compression/extension, which involves longitudinal deformation along the helical axis with spring-like behavior that allows for the storage of energy in the hydrogen bonding network. They can also bend sideways, twist their axis, and change their width by expanding and contracting.

Meanwhile, β-sheets exhibit distinct deformation patterns. These include in-plane stretching/compression through deformation within the plastic sheet and out-of-plane bending via flexural deformation perpendicular to the sheet plane. They also demonstrate shear deformation through the sliding of adjacent strands relative to each other and twisting via torsional deformation around the axis perpendicular to the sheet.

Furthermore, loop and turn structures present unique movements, such as stretching when the peptide backbone extends, changing position through angle adjustments, and folding or unfolding as they switch between organized and disorganized forms. These various deformation modes store energy differently. α-helices primarily store energy via hydrogen bonds and backbone torsional strain, while β-sheets store energy through hydrogen bond stretching and inter-strand interactions. Loops store energy mainly through dihedral angle strain and solvent interactions.

Energy absorbed from water collisions propagates through protein structures via several mechanisms. Collective vibrations involve low-frequency normal modes efficiently transmitting energy across long distances with minimal dissipation. In hydrogen bond networks, hydrogen bonds can break and reform one after another, passing energy along through a “domino effect.” Allosteric pathways consist of networks of dynamically coupled residues that efficiently transmit energy between distant sites. Solvent-mediated transfer involves water molecules located at protein interfaces, which help to transmit energy between structural elements. Electrostatic networks comprise charged and polar residue patterns, which propagate energy through long-range interactions.

Protein energy conversion processes operate across a hierarchy of structural levels, with complex interactions between them. At the local level (1–5 Å), individual residues absorb energy from water collisions, with surface-exposed residues serving as primary energy-capture sites. At the secondary structure level (5–15 Å), α-helices and β-sheets collect and store energy from multiple residues, acting as energy integration elements. At the domain level (15–30 Å), domains coordinate the energy stored in various secondary structures, channeling it toward functional sites. At the global level (>30 Å), large-scale conformational changes redistribute energy throughout the entire protein structure.

The efficiency of energy conversion depends on coordinated operation throughout the protein at all these levels. Proteins have evolved specific features at each level to optimize the flow of energy from surface collisions to catalytic sites. These features include flexible areas on the protein surface, secondary structures which enable better movement, connections between different parts of the protein that help to transfer energy more effectively, and catalytic sites located in such a way that energy can be gathered from various sources.

This framework helps us to understand how proteins work as systems that convert energy, combining different structural parts to channel random heat energy for use in specific chemical reactions. By taking in kinetic energy derived from water collisions, changing it into potential energy in their structure, and directing it to catalytic sites, proteins act as active energy converters instead of unchanging catalytic templates.

## 4. Secondary Structures as Energy Conversion Elements

### 4.1. Role of α-Helices in Energy Absorption and Conversion

α-helices are a common and well-researched part of proteins. These structures are spiral-shaped collections of amino acids, which are held together by hydrogen bonds between specific parts of amino acids that are four places apart in the chain. This regular, repeating structure creates a rigid yet flexible element which is critical for energy absorption and protein conversion.

The unique geometric properties of α-helices make them particularly efficient at absorbing and transmitting energy derived from water collisions. Their tube-like shape offers a large area to interact with solvent molecules, while the hydrogen bonds inside them help to spread and hold energy. When a water molecule collides with an α-helix, the impact energy can be absorbed through various deformation modes, including axial compression/extension, bending, twisting, and radial breathing.

Each deformation mode allows for the storage of potential energy that can be released to perform mechanical work or catalyze chemical reactions. The effectiveness of energy conversion in α-helices relies on factors such as the length of the helix, the types of amino acids it contains, and whether it includes stabilizing or destabilizing parts; for example, helices containing glycine residues (which lack a side chain) exhibit greater flexibility and can undergo larger deformations, potentially storing more energy. On the other hand, helices that have many alanine residues (which are good at forming helices) might be stiffer and better at moving energy more efficiently.

Molecular dynamics simulation studies have provided information about how α-helices respond to external forces. Miyashita et al. [12] demonstrated that helices can function as elastic springs, efficiently storing and releasing energy with minimal dissipation. This property makes them ideal candidates for energy conversion elements in enzymatic systems.

### 4.2. β-Sheets as Potential Energy Storage Systems 

β-sheets—the second type of primary protein structure—are made up of long chains of amino acids linked together by hydrogen bonds. This arrangement creates a pleated structure that can be parallel, antiparallel, or mixed, depending on the relative directions of the constituent strands. Like α-helices, β-sheets possess unique mechanical properties, making them effective energy storage systems within proteins.

The extended configurations of β-sheets provide several advantages for energy absorption and storage. Their flat shape offers a large area to interact with solvent molecules, increasing the chance of energy-moving collisions. The hydrogen bonds between adjacent strands act to distribute energy across the sheet, preventing localized strain and potential structural failure. β-sheets also exhibit mechanical anisotropy, allowing them to absorb and transmit energy along specific pathways in a selective manner.

Unlike α-helices—which primarily deform through compression/extension and bending—β-sheets can undergo shear deformation, in which adjacent strands slide relative to each other. This mode of deformation provides an additional mechanism for energy storage. When a water molecule hits a β-sheet, the energy from the impact can be absorbed in different ways, such as stretching or compression, bending up and down, or allowing the strands to slide past each other. Each of these modes stores energy differently: in-plane deformation mainly stretches the covalent bonds in the peptide backbone, while out-of-plane bending and shear deformation induce stress in the hydrogen bonds between strands.

Experimental and computational studies have demonstrated the remarkable mechanical properties of β-sheets. Rief et al. [14], using atomic force microscopy, showed that proteins rich in β-sheets can handle a great deal of mechanical force before they start to unfold, which means they can store a significant amount of potential energy. Similarly, by performing computer simulations, Gräter et al. [15] showed that β-sheets can effectively carry mechanical forces over long distances in a protein, thus helping to transfer energy.

### 4.3. Comparative Analysis of Energy Conversion Efficiency Among Secondary Structures

While both α-helices and β-sheets help to convert energy in proteins, they work in very different ways and have various levels of efficiency. Understanding these differences is essential when predicting how proteins harness Brownian motion to carry out catalytic functions.

Several metrics can be used to compare the energy conversion efficiency of different secondary structures. Energy density is the potential energy stored per unit volume or residue. Due to their more compact arrangement of amino acids, β-sheets typically have higher energy density than α-helices. Energy transmission efficiency represents the ability to transmit energy from the point of impact to distant regions of the protein with minimal dissipation. In this regard, α-helices often excel due to their coherent vibrational modes, which allow for the propagation of energy with low attenuation.

The response time reflects the speed at which a structure absorbs and releases energy. α-helices tend to respond more quickly due to their simpler deformation modes, while β-sheets may exhibit more complex, time-dependent responses. Directional specificity refers to the ability to channel energy along specific pathways. β-sheets demonstrate greater directional specificity due to their anisotropic mechanical properties, while α-helices typically distribute energy more uniformly.

Computational studies comparing the energy conversion properties of different secondary structures have yielded valuable insights. In their research, Leitner and colleagues [16] examined how energy moves in protein structures and found that α-helices transfer energy faster along their length than β-sheets. However, β-sheets showed more efficient energy transfer between adjacent strands, particularly when considering antiparallel arrangements.

Experimental evidence also suggests that the energy conversion efficiency of secondary structures depends on their local environment and connections to other protein elements. For example, α-helices connected to flexible loops may dissipate energy more rapidly than those anchored to rigid structural elements. Similarly, β-sheets embedded within the protein core may store energy more efficiently than those exposed to the solvent, as interactions with water molecules can dampen vibrational modes.

### 4.4. Super-Secondary Structures and Their Enhanced Energy Conversion Properties

Super-secondary structures—also called motifs or domains—are made up of combinations of α-helices, β-sheets, and connecting loops that are arranged in specific ways. These complex structures often improve how well their constituent parts convert energy, making them more effective at capturing and directing energy derived from random movement.

Common super-secondary structures with notable energy conversion properties include βαβ motifs, which are often observed in nucleotide-binding domains. These structures have a central α-helix with two parallel β-strands on the sides, forming a strong framework that can effectively direct energy from the outside to the center of the protein. α-helical bundles, which are composed of multiple α-helices arranged in parallel or antiparallel configurations, exhibit collective vibrational modes that can efficiently store and transmit energy, functioning as a coherent unit instead of as individual helices.

Barrels are closed structures formed by β-sheets wrapped into a cylinder. Their cylindrical geometry provides exceptional stability and efficient energy transmission around the barrel’s circumference. αα-corners, consisting of two α-helices connected by a short loop (often at approximately right angles), can serve as mechanical hinges, storing energy via bending motion between the helices.

The enhanced energy conversion properties of super-secondary structures arise from several factors. Cooperative deformation occurs when several secondary structures are linked, often increasing the overall ability to store energy in the design. The rigid connections between secondary elements in super-secondary structures serve to minimize energy losses through damping or friction. The specific arrangement of secondary structures can create preferred pathways for energy transmission, directing energy from impact points to catalytic sites. Super-secondary structures can also vibrate in ways that improve the absorption of energy at specific frequencies, making them better suited to random movement patterns in fluids.

Research on enzyme dynamics has revealed the importance of super-secondary structures in catalysis. Hammes-Schiffer and Benkovic [17] showed that groups of connected movements across different secondary structures are crucial for enzymes such as dihydrofolate reductase to work correctly. Similarly, Henzler-Wildman and others [9] performed research on adenylate kinase and showed that the movements of different super-secondary structures are closely linked to how the enzyme functions during its catalytic cycle.

The concept of proteins as dynamic energy converters is particularly evident in the behavior of super-secondary structures. These complex structures, developed over billions of years, effectively capture the random energy derived from Brownian motion and use it to perform specific modifications that promote catalysis. Understanding the unique energy conversion properties of different super-secondary structures offers helpful information about how proteins function as molecular machines rather than static templates.

## 5. Catalytic Domains and Their Structural Dynamics

### 5.1. Relationship Between Domain Deformation and Catalytic Activity

The connection between protein domain deformation and catalytic activity represents a fundamental aspect of enzyme function that has been increasingly recognized in recent years. Catalytic domains—the parts of enzymes where substrates attach and chemical changes happen—were once thought to be stable structures that simply held catalytic residues in the right place. However, mounting evidence suggests that the dynamic properties of these domains are essential for their catalytic function.

The deformation of catalytic domains under the influence of Brownian motion serves multiple purposes in enzyme catalysis. Domain changes can shift the exact placement of catalytic residues, changing the shape of the active site to improve how it interacts with the substrate at various stages of the catalytic cycle. Deformation can create tension within the catalytic domain that can be passed on to the substrate, weakening certain bonds and reducing the energy needed for the reaction. Dynamic fluctuations allow the enzyme to cycle through multiple conformations, increasing the probability of achieving a catalytically competent state. Domain movements can help to release products after the chemical reaction, preventing product buildup and allowing the enzyme to operate more effectively.

Experimental evidence supporting the relationships between domain deformation and catalytic activity has been obtained using various techniques. For example, research using hydrogen–deuterium exchange mass spectrometry has shown that parts of enzymes that can change shape easily are often linked to areas which are critical for chemical reactions. In the same way, NMR relaxation experiments have shown that the rate at which enzyme domains can change shape usually aligns with how fast they carry out their chemical reactions, indicating a direct link between the movement of these domains and their ability to catalyze reactions.

Computational studies have offered additional details about such relationships. Molecular dynamics simulations performed by Bhabha et al. [18] on dihydrofolate reductase showed that the enzyme changes shape in specific ways during chemical reactions, allowing it to better bind to the substrate, carry out the chemical modification, and release the product. These shape changes were considered to be essential for effective catalysis, as mutations that changed how the domain moved greatly impacted the enzyme’s activity.

### 5.2. Substrate Binding in Dynamic vs. Static Models

How a substrate attaches to an enzyme is an integral part of how enzymes work, emphasizing the differences between dynamic and static models of protein function. In traditional static models, substrate binding is usually explained by Fischer’s “lock and key” model or Koshland’s “induced fit” hypothesis, which focus on how well the shapes of the enzyme and substrate fit together. However, these models fail to fully account for the dynamic nature of the enzyme and the binding process.

In the dynamic model of substrate binding, several key features emerge. Instead of causing a change when they attach, substrates might connect with one or more of the different shapes that the enzyme already possesses due to random movement. This concept, known as “conformational selection,” has gained substantial support through experimental and computational studies. The path by which a substrate enters the active site is not static, instead involving a series of transient interactions and conformational adjustments as the substrate navigates through the protein.

Active sites are not rigid structures but fluctuate continuously under the influence of Brownian motion, alternately exposing and concealing potential binding surfaces. When a substrate binds, it changes the energy setup of the protein, affecting how likely it is to adopt certain different shapes and possibly helping with the following steps in the chemical reaction.

Experimental evidence supporting dynamic models of substrate binding has been obtained using various techniques. Single-molecule FRET studies have shown how enzymes change shape when they bind to substrates, uncovering that several states allow for effective binding to the substrate. Similarly, NMR experiments have demonstrated that substrate binding often shifts the equilibrium between pre-existing conformational states rather than inducing entirely new conformations.

The concept that substrates preferentially bind to proteins in high-energy states, proposed by Cao and Ding [7], represents an intriguing extension of the dynamic binding model. In this context, the movement of protein parts due to random motion creates temporary high-energy shapes that reveal binding sites or make it easier for substrates to attach. Once bound, the substrate may stabilize such a high-energy conformation, effectively capturing a portion of the potential energy stored in the deformed protein structure.

### 5.3. Catalytic Mechanisms Driven by Protein Deformation

The transformation of potential energy into catalytic action in deformed protein structures is a key part of the dynamic energy conversion model. This energy-driven catalysis can operate through various mechanisms, depending on the specific reaction and enzyme involved.

One way in which changes in protein shape can facilitate catalysis is through direct mechanical force; here, the strain due to the protein’s deformation affects specific bonds in the substrate, pushing them into a shape that makes it easier for the reaction to occur and reducing the energy needed to start the reaction. Electrostatic field modulation occurs when movements in the protein change the positions of charged and polar groups in the active site, which alters the electric field around the substrate and helps to stabilize transition states or intermediates.

Dynamic hydrogen bonding networks represent another mechanism. Deformation can change how hydrogen bonds are arranged in an enzyme, forming temporary patterns that enable proton transfer or support charged transition states. Movements in the protein can allow for the placement of water molecules exactly where they need to be for certain chemical reactions, thus speeding up the corresponding actions. Movements in the protein can also affect how far apart the atoms involved in hydrogen transfer are, making it more likely for quantum tunneling to occur and speeding up reactions.

The energy needed for these catalytic processes comes from the potential energy captured in the twisted protein structure, which is created by the movement of colliding water molecules. This energy conversion pathway provides a mechanistic explanation for how enzymes can lower activation energy barriers without violating thermodynamic principles.

Experimental evidence supporting deformation-driven catalysis has been obtained from various sources. Research on “heavy” enzymes—in which non-exchangeable hydrogen atoms are swapped with deuterium or tritium—has shown that lowering the vibration speed of the protein structure can significantly reduce how fast the enzyme works, even if the electrical properties stay the same. This suggests that the dynamic properties of the protein directly contribute to catalysis rather than the mere positioning of catalytic residues.

Similarly, research on how temperature affects enzyme activities has often shown that enzymes work best at certain temperatures at which the benefits of increased movement (which helps in changing the enzyme’s shape) are balanced with the need for the protein to stay stable. This observation aligns with the concept that domain deformation provides energy for catalysis, with the optimal temperature representing a balance between increased deformation energy and structural stability.

## 6. Experimental Evidence and Methods

### 6.1. Experimental Toolbox for Studying Protein Dynamics

Understanding protein dynamics across multiple timescales requires a diverse toolkit of experimental techniques. Each method provides unique insights into different aspects of the motion of proteins, from rapid local fluctuations to slow global conformational changes which are relevant to catalysis. Below, we organize these techniques by category and provide standardized information regarding their capabilities and limitations.

Time-resolved X-ray crystallography employs ultrashort X-ray pulses to capture protein structures at specific time points following the initiation of a reaction. It operates on timescales ranging from nanoseconds to seconds and can achieve a spatial resolution of 1.5–3.0 Å, offering insights into conformational changes during catalysis and the formation of reaction intermediates. However, this method requires proteins that can be crystallized, may introduce potential crystal packing artifacts, and typically necessitates photochemical triggering. Serial femtosecond crystallography (SFX) uses X-ray free electron lasers to collect diffraction data from microcrystals before radiation damage occurs, working on femtosecond to picosecond timescales with a spatial resolution of 1.5–3.0 Å. In this way, it can reveal ultrafast structural changes and bond formation/breaking events. However, it requires highly specialized facilities, is characterized by high sample consumption, and involves complex data processing.

In time-resolved cryo-electron microscopy (cryo-EM), structural snapshots of proteins in solution are captured by rapid freezing at different reaction time points. This technique operates on millisecond to minute timescales with a spatial resolution of 2.5–4.0 Å, allowing for the observation of large-scale conformational changes and macromolecular assemblies; however, it has a lower resolution than crystallography, has complex sample preparation requirements, and involves computationally intensive processing. Small-angle X-ray scattering (SAXS) measures the scattering of X-rays by proteins in solution to determine their overall shape and size changes, functioning on millisecond to minute timescales with a spatial resolution of 10–50 Å (low resolution) and revealing global conformational changes, protein flexibility, and oligomerization. However, it has low resolution, provides limited structural detail, and requires ensemble measurements.

Nuclear magnetic resonance (NMR) spectroscopy involves measuring the magnetic properties of atomic nuclei to probe protein structures and dynamics in solution, covering picosecond to second timescales with atomic resolution for assigned resonances. While this approach provides information on site-specific flexibility, conformational exchanges, local unfolding, and allosteric networks, it has size limitations (~50 kDa), it requires complex data analysis, and isotopic labeling is needed. Fluorescence spectroscopy monitors changes in the fluorescence properties of natural or introduced fluorophores during the motion of proteins, covering nanosecond to minute timescales with a spatial resolution of 10–100 Å between labeled sites. In this way, domain movements, binding events, and conformational changes can be observed; however, the introduction of a fluorophore is necessary, which may perturb the protein’s dynamics.

In electron paramagnetic resonance (EPR) spectroscopy, the spin labels of unpaired electrons are detected to monitor distances and mobility. This approach operates at nanosecond to millisecond timescales with a spatial resolution of 5–80 Å between spin labels, revealing site-specific dynamics, distance changes, and local mobility. However, it requires site-specific spin labeling and may cause perturbation of the protein’s properties.

Vibrational spectroscopy (IR, Raman) involves measuring the vibrational modes of chemical bonds to detect structural changes, which operates at femtosecond to nanosecond timescales with chemical group-specific spatial resolution. In this way, bond vibrations, hydrogen bonding changes, and secondary structure dynamics can be observed; however, this approach involves complex spectral interpretation, may result in overlapping signals, and often requires specialized labeling. Single-molecule FRET (smFRET) measures distances between fluorophore pairs on individual protein molecules, which operating at millisecond to minute timescales with a spatial resolution of 10–100 Å between labeled sites. This approach can reveal conformational distributions, transition pathways, rare events, and dynamic heterogeneity; however, it requires dual-labeling and imposes photobleaching limits on the observation time as well as distance range constraints. Optical tweezers involve the use of focused laser beams to trap and manipulate single molecules, allowing for the measurement of forces and displacements, and function at millisecond to minute timescales with a spatial resolution of 0.1–1 nm displacement. They can provide information on mechanical properties, unfolding/refolding pathways, and energy landscapes; however, this approach requires tethering to beads, has limited force resolution, and typically probes non-equilibrium processes.

In atomic force microscopy (AFM), a nanoscale tip is used to probe the surface topography and mechanical properties of molecules. This approach operates at millisecond to minute timescales with a spatial resolution of 0.5–5 nm, revealing surface topography changes, mechanical stability, and unfolding pathways. However, it requires surface-bound samples, may introduce surface artifacts, and has limited time resolution. In high-speed AFM, the surface topography is rapidly scanned to create “molecular movies” of protein motion, with a timescale capability of ~100 milliseconds per frame and a spatial resolution of 1–3 nm, allowing conformational changes, molecular interactions, and assembly/disassembly to be visualized. However, it requires surface-bound samples and is limited to observing surface-accessible dynamics.

Hydrogen–deuterium exchange mass spectrometry (HDX-MS) measures the rate of exchange between hydrogen and deuterium to probe protein dynamics and solvent accessibility, operating at second to hour timescales with a spatial resolution of peptide fragments (5–20 residues). This approach provides information on regional flexibility, solvent accessibility, binding interfaces, and allosteric effects; however, it has limited spatial resolution, requires complex data analysis, and has back-exchange issues.

In NMR hydrogen exchange, NMR is used to detect the exchange of individual backbone amide hydrogens with deuterium, which functions at minute to day timescales with a single-residue spatial resolution. This helps to reveal site-specific conformational dynamics, local unfolding, and hydrogen bond stability; however, it requires large quantities of isotopically labeled proteins and is limited to observable NMR signals.

Molecular dynamics (MD) simulations numerically solve Newton’s equations of motion for all atoms in a system over time, operating at femtosecond to microsecond timescales (up to milliseconds with specialized hardware) with atomic detail spatial resolution, enabling the analysis and visualization of atomic motions, conformational changes, energy landscapes, and water interactions. However, they utilize force field approximations, operate at limited timescales, have high computational cost, and require validation.

Standard mode analysis (NMA) involves calculating the principal vibrational modes of proteins by diagonalizing the Hessian matrix, which is not explicitly time-dependent and focuses more on collective motions, having atomic to coarse-grained spatial resolution. In this way, collective motions, domain movements, flexible regions, and mechanical coupling can be revealed; however, this approach uses a harmonic approximation, typically allows for near-equilibrium analysis only, and requires minimal static energy.

Markov state models (MSMs) are kinetic models of conformational transitions constructed from simulation data, with timescale capability that can extend upon simulation timescales by orders of magnitude and spatial resolution, depending on underlying simulation data. Such models provide information on metastable states, transition pathways, kinetic rates, and equilibrium populations. However, they require extensive sampling, pose challenges related to state definition, and are complex to validate. Machine learning approaches use data-driven algorithms to identify patterns and make predictions about protein dynamics, with variable timescale capability and spatial resolution depending on the training data and model design used. The developed models allow for feature extraction, dimensional reduction, the prediction of dynamics, and pattern recognition. However, their performance is dependent on the quality of the training data used, and they may suffer from interpretability challenges and potential overfitting. Each of these techniques provides a different perspective on protein dynamics, and their integration is essential for developing a comprehensive understanding of how proteins harness Brownian motion for catalysis, with the selection of appropriate methods depending on the specific aspect(s) of protein dynamics being investigated, the timescale(s) of interest, and the system under study.

### 6.2. Evidence from X-Ray Crystallography and Cryo-Electron Microscopy

Although traditionally associated with static structures, advanced X-ray crystallography and cryo-electron microscopy (cryo-EM) applications have provided valuable evidence supporting the dynamic energy conversion model of protein function.

One of the most compelling lines of evidence comes from the observation of multiple conformational states in crystal structures and cryo-EM reconstructions of the same protein. Fraser et al. [19] used X-ray crystallography at room temperature to find different shapes of proteins that could not be seen in frozen structures, demonstrating a pattern of movements that spread throughout the protein. Cryo-EM research performed by Nakane et al. [20] on the SARS-CoV-2 spike protein revealed several different protein shapes in the same sample, showing that the protein can change shape even when very cold. The “molecular movies” made using time-resolved crystallography have shown that proteins change shape in real time, proving that proteins are active and flexible in how they work.

These findings suggest that proteins are not stiff and unchanging but, instead, are flexible and can take on different shapes due to random movements.

Comparing protein structures determined at different temperatures has provided information regarding their energy absorption and storage mechanisms. Structures at higher temperatures usually show more chaotic structures in the loop areas and parts of the protein exposed to the surface, which aligns with these areas taking in energy from the extra heat. The progressive “melting” of protein structures with increasing temperature often begins in specific regions, which may represent energy-absorbing elements that protect the core structure. Keedy et al. [21] demonstrated that proteins exhibit a hierarchy of motions that are activated at different temperatures, suggesting a coordinated response to increasing energy input.

These temperature-dependent effects align with the model of proteins as systems that absorb, store, and utilize energy from their environment. The temperature factors (B-factors) in crystal structures—which show how much atoms move—provide clues about the parts of proteins that might help to absorb and store energy. Secondary structures with high B-factors often match areas that change shape when the protein functionally operates, indicating they might act as energy storage parts. Groups of amino acids with similar B-factors usually link areas on the surface of proteins to their active sites, which might indicate how energy moves from areas where water collisions occur to the protein’s active sites. Observation of B-factor patterns in similar proteins has revealed that flexible areas are often preserved through evolution, indicating these dynamic features are functionally important.

Although B-factors are affected by more than just heat movement, their patterns provide important hints about how proteins move and how they might convert energy.

### 6.3. NMR Studies Supporting Dynamic Catalytic Models

NMR spectroscopy has been very useful for examining how proteins move in solution, proving that Brownian motion is essential for how proteins work and facilitate chemical reactions.

NMR relaxation dispersion experiments have revealed the presence of “invisible” excited states in many proteins, including enzymes, during catalysis. Research by Boehr et al. [22] on dihydrofolate reductase demonstrated that the enzyme presents conformations resembling various catalytic intermediates even in the absence of substrates, suggesting that these conformational changes are intrinsic to the protein rather than induced solely by substrate binding. NMR investigations of cyclophilin A by Eisenmesser et al. [23] revealed that the rate of conformational exchange matched the catalytic rate, providing strong evidence that protein dynamics are directly coupled to catalytic function. Recent improvements in CEST (chemical exchange saturation transfer) and CPMG (Carr–Purcell–Meiboom–Gill) experiments have made it possible to observe rare, excited states that could be critical energy-rich shapes needed for catalysis.

These observations support the model of proteins as dynamic entities that constantly adopt different conformational states due to Brownian motion, with certain states facilitating various steps in the catalytic reaction.

NMR studies at different temperatures have provided insights into how proteins respond to increased thermal energy. Kay and colleagues [24] demonstrated that proteins exhibit a hierarchy of dynamic processes across different timescales, with faster motions being activated at lower temperatures and slower motions with larger amplitude requiring higher thermal energy. The temperature-dependent conformational exchange rates often present as non-Arrhenius behaviors, suggesting complex energy landscapes with multiple barriers and states. Studies of heat-loving enzymes show that they are usually less flexible at room temperature than their moderate-temperature counterparts. Still, they have similar flexibility at their optimal working temperatures, indicating that the right amount of movement is essential for their function.

These temperature-dependent effects align with the model of proteins utilizing thermal energy from their environment to drive conformational changes which are necessary for their functioning.

NMR experiments that track the flow of energy through protein structures have provided evidence for specific pathways that connect surface regions to catalytic sites. Studies using isotope-edited NMR to follow how energy moves within proteins have revealed groups of connected amino acids that effectively pass energy through protein structures. Research on allosteric proteins has shown that groups of connected residues link allosteric sites to active sites, showing that proteins can communicate over long distances through certain movements. Recent developments in high-pressure NMR have allowed researchers to highlight how proteins respond to mechanical stress, revealing regions that preferentially absorb and transmit energy.

These observations support the concept of proteins as networks of coupled elements that can transmit energy from the surface—namely, where water collisions occur—to internal sites where catalysis occurs.

### 6.4. Single-Molecule Studies of Protein Fluctuations During Catalysis

Single-molecule techniques have revolutionized our understanding of protein dynamics by removing the need for ensemble averaging, thus revealing the heterogeneous behaviors of individual molecules. These approaches provide compelling evidence for the roles of conformational dynamics in enzyme catalysis.

Single-molecule FRET (smFRET) experiments have allowed for the direct visualization of the conformational changes that enzymes undergo during catalysis. Henzler-Wildman et al. [9], using smFRET, showed that adenylate kinase moves between open and closed shapes even when there is no substrate, and the speed of this shape change matches with how fast it catalyzes reactions. Research on the ribosome by Blanchard and colleagues [25] demonstrated that this molecular machine presents different shapes while making proteins, with certain shape changes linked to specific steps in the process. Recent improvements in time-resolved smFRET have allowed scientists to monitor changes in protein shapes with millisecond precision, thus revealing the order and timing of events during catalytic cycles.

These direct observations of how proteins change shape strongly suggest that proteins are active and changeable rather than fixed structures, with the energy for these shape changes likely deriving from random movement.

Single-molecule studies have revealed surprising heterogeneity in the behaviors of supposedly identical enzyme molecules. Lu et al. [26] performed research on cholesterol oxidase and revealed that individual enzyme molecules exhibit different catalytic rates and fluctuate between high- and low-activity periods, a phenomenon known as “dynamic disorder.” Tan and Rief [27] used optical tweezers to show that individual protein molecules take different paths when folding and have different mechanical properties, highlighting the natural differences in how proteins behave. Single-molecule experiments on various enzymes have shown that the catalytic rates of individual molecules fluctuate over time, suggesting changes in the conformational dynamics of proteins.

This observed variation matches the idea that proteins are active systems that are affected by random interactions with water molecules, with the unpredictable movement caused by Brownian motion leading to differences in how they behave.

Advanced single-molecule techniques have enabled direct measurement of the energy landscapes that govern protein conformational changes. Optical tweezer experiments conducted by Bustamante and colleagues [28] allowed for the determination of the energy paths involved in the folding and conformational change of proteins, highlighting the obstacles and temporary states during these processes. In magnetic tweezer studies, the mechanical properties of proteins under force have been measured, revealing how proteins store and release energy during conformational changes. Recent advances in high-speed AFM have allowed scientists to visualize how proteins move and change shape at the microscopic scale, revealing the changes in structure that occur as they function.

These direct measurements of energy landscapes and mechanical properties offer information about how proteins store and utilize energy derived from Brownian motion, supporting the model of proteins as dynamic energy converters.

The experimental evidence from these various techniques strongly supports the dynamic energy conversion model of protein functions. The different shapes and movements seen in structural studies and the direct observation of these changes in single-molecule experiments all indicate that proteins are active and use energy from their surroundings to change shape, which is essential for their function. This growing body of evidence challenges the traditional view of proteins as static templates, highlighting the vital role of dynamics in the function and catalytic ability of proteins.

## 7. Computational Approaches to Protein Dynamics

### 7.1. Molecular Dynamics Simulations of Protein–Water Interactions

Molecular dynamics (MD) simulations have emerged as one of the most powerful computational tools for studying protein–water interactions and their roles in the dynamics and functioning of proteins. These simulations numerically solve Newton’s equations of motion for all atoms in a system, providing detailed trajectories that reveal how proteins respond to collisions with water molecules and how the resulting energy is distributed throughout the protein structure.

Explicit solvent MD simulations include individual water molecules, allowing for direct observation of protein–water interactions. These simulations show how water molecules hit proteins, which helps us to determine how this impact changes the protein’s shape (both in local areas and overall). Cerutti et al. [29] demonstrated that these collisions generate a spectrum of protein motions, from localized vibrations to large-scale domain movements. Researchers can identify specific pathways in which energy propagates through protein structures by tracking the flow of energy following water collisions. Sharp and Skinner [30] revealed that energy transfer is not uniform but rather follows preferred routes defined by the protein’s structure and dynamic properties.

Explicit solvent simulations have revealed that water molecules transfer momentum to proteins and actively participate in conformational transitions by forming and breaking hydrogen bonds with the protein. Fenwick et al. [31] demonstrated that these water-mediated processes can significantly lower energy barriers for conformational changes. The behavior of water molecules near proteins (i.e., the hydration shell) influences the efficiency of energy transfer. Halle [32] revealed that hydration shell water exhibits distinct dynamical properties when compared to bulk water, with implications for how effectively Brownian motion can drive protein dynamics.

Recent improvements in computational power and algorithms have allowed for simulations using explicit solvents that run for much longer—from nanoseconds to microseconds or even longer—making it possible to observe slow changes in protein shape that are important for catalysis. For example, Shaw and his team [33] ran simulations of proteins in an explicit solvent that lasted milliseconds, revealing complicated folding processes and functional behaviors that could not be captured through shorter simulations.

Various enhanced sampling methods have been developed to overcome the timescale limitations of conventional MD simulations. Accelerated molecular dynamics (AMD) makes it easier for the simulation to move past energy barriers, allowing the system to more often explore rare changes in shape. Grant et al. [34], using AMD, observed hidden binding sites in enzymes that can only be reached through specific protein movements. Replica exchange molecular dynamics (REMD) runs several copies of the system at various temperatures and swaps their setups occasionally, which helps to explore different system shapes more effectively. Sugita and Okamoto [35] demonstrated that this approach can reveal the complete ensemble of conformational states accessible to a protein. Metadynamics approaches use special forces based on past events to help the system explore areas of conformational space that have not been sampled. Laio and Parrinello [36] showed that metadynamics helps to efficiently map complex free energy landscapes governing protein dynamics.

These improved sampling methods are beneficial for understanding how proteins leverage Brownian motion to help with chemical reactions, as they can reveal rare high-energy shapes that are important for storing and converting energy.

Specialized analysis methods have been developed to extract information about the flow and storage of energy through MD simulations. Standard mode analysis (NMA) involves the decomposition of protein motions into vibrational modes, revealing the collective motions that help to most efficiently store and transmit energy. Bahar and colleagues [37] showed that significant catalysis movements usually match low-frequency normal modes, indicating that evolution has fine-tuned these movements to more effectively utilize energy. Principal component analysis (PCA) allows for the determination of the main movements in MD trajectories, indicating how proteins react to and use thermal energy. Amadei et al. [38] demonstrated that a few “essential modes” often dominate protein dynamics, potentially acting as primary energy conduits.

Energy decomposition methods measure how much different interactions (e.g., bonded, electrostatic, and van der Waals) contribute to the total energy, revealing how energy is held in different parts of the structure. Kannan and Vishveshwara [39] identified networks of interactions that efficiently store and transmit energy within protein structures. Methods that use information theory (e.g., mutual information and transfer entropy) can reveal statistically linked residues which allow for the creation of networks for the transfer of energy and information. McClendon et al. [40] revealed that these networks often connect surface-exposed regions (i.e., where water collisions occur) to catalytic sites.

Together, these computational approaches have provided detailed insights into how proteins interact with water molecules, absorb energy from collisions, and utilize this energy to perform conformational changes that drive catalysis.

### 7.2. Brownian Dynamics Models for Protein Motion

While molecular dynamics simulations provide atomic-level detail, Brownian dynamics (BD) models offer a complementary approach that is particularly well-suited for studying how proteins respond to the stochastic forces of water molecules over longer timescales. Through explicitly modeling the random forces arising from water collisions without representing individual water molecules, BD simulations can access timescales which are relevant to many catalytic processes while focusing on the protein’s response to Brownian motion.

BD simulations are based on the Langevin equation, which describes the motion of particles under the influence of random forces:

m(d^2^r/dt^2^) = F(r) − γ(dr/dt) + R(t)

where m is the particle’s mass, r is its position, F(r) is the systematic force derived from the potential energy function, γ is the friction coefficient, and R(t) is a random force representing thermal fluctuations due to solvent collisions.

In the overdamped limit—which is often appropriate for proteins in solution—inertial effects become negligible and the equation simplifies to

dr/dt = F(r)/γ + √(2kBT/γ)ξ(t)

where Ξ (t) is Gaussian white noise, kB is Boltzmann’s constant, and T is temperature.

BD simulations have been applied to various aspects of protein dynamics and functions. They can accurately model the diffusion of proteins in solution, capturing how Brownian motion influences their translational and rotational movement. Elcock and his team [41] showed that BD simulations can reliably mimic how proteins move in experiments, confirming that this method is good for studying Brownian effects. Incorporating flexible protein models, BD simulations can capture how random forces due to water collisions drive conformational changes. Spiriti and Zuckerman [42] found that BD simulations using simpler protein models help to effectively explore the different shapes proteins can take while still accurately capturing how Brownian motion affects them.

BD simulations are particularly valuable for studying how Brownian motion influences the association of proteins, which is often a key step in enzymatic processes. Gabdoulline and Wade [43] showed that BD simulations can accurately estimate how quickly protein complexes come together, considering the effects of electric charges and how they move toward each other. Through modeling the diffusional encounter between enzymes and substrates under the influence of Brownian motion, BD simulations offer information about how random thermal forces influence the early stages of enzymatic reactions. Wade et al. [44] showed that the electric fields around enzymes can help to guide substrates to their active sites, making the random encounters driven by Brownian motion more effective.

BD simulations are often integrated with other computational approaches to provide a more complete picture of protein dynamics. Hybrid MD/BD approaches use MD simulations for areas where detailed atomic information is essential (e.g., active sites) and BD for areas where a simpler model suffices. Erban [45] showed that these hybrid methods can effectively track protein movements at different levels while considering the effects of Brownian motion. Advanced BD methods incorporate internal protein dynamics, allowing the protein structure to respond to Brownian forces. Ando and Skolnick [46] showed that these approaches can effectively model how stochastic collisions with solvents influence the conformational states of proteins.

BD simulations that include hydrodynamic interactions can reveal how the movement of one part of a protein affects the flow of solvent around other parts, providing a better picture of how Brownian forces impact the complex movements of proteins. Frembgen-Kesner and Elcock [47] showed that adding hydrodynamic interactions greatly enhances the accuracy of BD simulations when predicting how proteins move and interact. Combining BD simulations with methods to calculate free energy landscapes helps to clarify how Brownian motion enables proteins to explore different conformational states. Rojnuckarin et al. [48] used this approach to investigate how thermal fluctuations allow proteins to overcome energy barriers between functional states.

Brownian dynamics simulations offer a helpful way to study how proteins leverage random movements caused by water collisions to perform their functions and catalyze reactions. Through explicit modeling of the stochastic nature of these interactions while abstracting away the explicit representation of water molecules, BD approaches enable the study of protein dynamics on timescales which are relevant to many biological processes.

### 7.3. Integration of AlphaFold Predictions with Dynamic Models

The recent revolution in protein structure prediction driven by AI methods—particularly DeepMind’s AlphaFold—has opened new opportunities for studying protein dynamics and function. While AlphaFold and similar tools primarily predict static structures, their integration with dynamic modeling approaches offers promising avenues for understanding how proteins harness Brownian motion for catalysis.

AlphaFold has demonstrated remarkable accuracy in predicting protein structures but has significant limitations in terms of capturing protein dynamics. AlphaFold usually provides just one fixed structure that shows the most stable form, without including the different shapes that proteins can take due to Brownian motion. Raisinghani et al. [49] noted that this limits its ability to directly capture the dynamic properties that are essential for understanding catalysis.

The confidence scores for each part of the protein (pLDDT) given by AlphaFold often relate to more flexible or disordered areas, which can provide clues about how the protein moves. Akdel et al. [50] demonstrated that regions with low pLDDT scores frequently correspond to functionally critical flexible segments. While AlphaFold generally does not predict alternative conformational states that may be critical for catalysis, it sometimes produces multiple slightly different models that hint at conformational flexibility. Ohnuki and Okazaki [51] suggested that analyzing variations in multiple AlphaFold predictions can offer clues about potential conformational transitions.

The training data for AlphaFold was mainly derived from crystallographic structures, which usually show proteins in their most stable forms instead of the various shapes they may take in solution. This leads to a systematic bias toward static representations of proteins.

Despite these limitations, integrating AlphaFold predictions with dynamic modeling approaches has emerged as a powerful strategy for studying protein functions.

Several approaches have been developed to combine AlphaFold predictions with dynamic modeling methods. Using structures predicted by AlphaFold as starting points for molecular dynamics simulations allows researchers to investigate how proteins behave over time, even when they do not have experimental structures. Heo and Feig [52] demonstrated that MD simulations initiated using AlphaFold models can accurately reproduce the dynamic properties observed in experimentally characterized proteins.

Multiple sequence alignments (MSAs) provide evolutionary information that contains signals about protein dynamics and structure, forming the basis for AlphaFold predictions. Lu et al. [53] found that the patterns of mutations seen in MSAs often relate to groups of amino acids that play a role in energy transfer and conformational changes. Through analyzing local energetic frustration in AlphaFold-predicted structures, researchers can identify regions that are likely to undergo conformational changes. Neelamraju et al. [54] demonstrated that integrating frustration analysis with AlphaFold predictions allows for the successful prediction of protein conformational motions, which reveals how proteins harness Brownian motion to perform certain functions.

Various methods have been developed to generate conformational ensembles, starting with AlphaFold predictions. Degiacomi et al. [55] found that normal mode analysis of AlphaFold structures allows one to effectively create groups of protein shapes that reflect how proteins move while functioning. Comparing AlphaFold predictions for homologous proteins, researchers can identify conserved flexible regions that may be functionally important. Bryant et al. [56] demonstrated that such comparative analyses can reveal dynamic features that are conserved across protein families.

Several recent studies have successfully integrated AlphaFold predictions with dynamic modeling to offer conclusions about protein functions. Ohnuki and Okazaki [51] used AlphaFold predictions along with a faster molecular dynamics approach to determine missing shapes of transporter proteins, showing how these proteins move between different forms during transport using Brownian motion. Gupta et al. [57] used AlphaFold predictions combined with elastic network models to investigate the collective motions of enzyme domains, revealing how these motions contribute to enzyme catalysis.

Fu and Zhang [58] demonstrated that combining AlphaFold predictions with replica exchange molecular dynamics allows for effective sampling of the conformational space of partially disordered proteins, revealing how these proteins utilize their dynamic properties to function. Lu et al. [53] combined AlphaFold predictions with energy landscape analysis to discover allosteric pathways in proteins, demonstrating how changes in shape move through protein structures to affect their functions.

These examples demonstrate how combining AlphaFold predictions with dynamic modeling can help us to better understand how proteins work rather than just considering their fixed shapes.

## 8. Implications for Drug Design and Enzyme Engineering

### 8.1. Limitations of Structure-Based Drug Design Using Static Models

Traditional structure-based drug design (SBDD) methods mainly use fixed protein structures, typically created via X-ray crystallography or cryo-electron microscopy. While these approaches have yielded numerous successful drugs, they suffer from fundamental limitations arising from their neglect of protein dynamics; particularly the role of Brownian motion in generating functionally important conformational states.

Static models often fail to capture the dynamic nature of binding sites, leading to several limitations. Many proteins contain “cryptic pockets” that are not visible in static structures but emerge dynamically through conformational changes driven by Brownian motion. Cimermancic et al. [59] estimated that over 80% of proteins contain such cryptic sites, representing untapped drug discovery opportunities. Traditional SBDD approaches based on static structures typically miss these potential binding sites.

Static models represent binding sites in a single conformation, failing to capture the ensemble of states that proteins may adopt in solution. This can result in inflexible ligands that work well with the fixed structure seen in crystals but which do not fit well with the changing forms of proteins in the body. Static structures often include only a few structural water molecules, neglecting the dynamic water network that continuously exchanges with bulk solvents due to Brownian motion. Such a dynamic water network can play crucial roles in ligand binding, as shown by Ladbury [60], and its neglect can ultimately lead to sub-optimal drug design outcomes.

The dynamic energy conversion model has important implications for understanding and predicting binding affinities. Traditional methods often have difficulty in accurately considering the energy changes related to binding, which are closely connected to how proteins move. Chodera and Mobley [61] demonstrated that neglecting protein dynamics can lead to systematic errors in binding affinity predictions.

The time a drug spends bound to its target (i.e., its residence time) often correlates better with efficacy than its equilibrium binding affinity. Copeland et al. [62] showed that the residence time is intimately connected to protein dynamics, with drugs that effectively modulate protein dynamics often exhibiting longer residence times. Static models offer very little information about these kinetic aspects of drug binding. By concentrating on active sites or specific binding areas, static methods often miss other important sites where molecules could change how proteins function by affecting their dynamics. Guarnera and Berezovsky [63] demonstrated that such allosteric sites are common in proteins and represent valuable targets for drug discovery.

Drug resistance often emerges through mutations that alter protein dynamics rather than directly affecting drug binding. In this context, mutations far from the binding sites can change how a protein moves and functions, affecting how well a drug binds or works even if the binding site itself does not change. Whitford et al. [64], in their research on HIV protease, showed that many mutations that cause resistance do so by changing how the protein moves instead of just blocking the inhibitor from binding. Proteins can adapt to inhibitors through compensatory changes in their dynamics, maintaining function despite drug binding. Static models fail to capture these adaptive responses, limiting their utility for the design of drugs that can overcome resistance.

In some cases, resistance arises from shifts in the population of different conformational states rather than changes in the structures associated with individual states. Bowman and Geissler [65] showed that such population-shift mechanisms are common but may be invisible when considering static structural approaches.

The limitations of static models indicate the importance of approaches that explicitly account for protein dynamics and the role of Brownian motion in generating functionally relevant conformational states. Through incorporating these dynamic aspects, drug designers can potentially access a broader range of binding sites, design ligands that more effectively modulate protein functions, and develop strategies to overcome resistance.

#### Strategies for Targeting Dynamic Catalytic States

Viewing proteins as flexible energy converters that utilize Brownian motion to enable their chemical reactions creates new chances for drug design and blocking enzymes. Several innovative strategies have emerged for targeting proteins that are based on their dynamic properties rather than simply their static structures.

Drugs can more effectively modulate protein functions by targeting entire conformational ensembles rather than single structures. The multiple receptor conformations (MRC) approach uses different shapes of proteins—which can be derived from experimental data, MD simulations, or tools like AlphaFold with sampling methods—to represent the variety of shapes that the target can take. Amaro et al. [66] demonstrated that MRC approaches significantly improve virtual screening success rates when compared to single-structure methods.

Weighted ensemble approaches consider how likely the different shapes of a protein are, enabling designers to focus on essential shapes based on their functions instead of how common they are. Bowman et al. [67] showed that such approaches can help to identify ligands that selectively bind to specific functionally relevant states, even if they are rarely populated. Through creating Markov state models (MSMs) derived from molecular dynamics (MD) simulations, researchers can find important stable states and design ligands that specifically attach to these states or influence the changes between them. Plattner and Noé [68] demonstrated that MSM-based approaches can reveal druggable conformational states that may be missed by traditional methods.

The dynamic energy conversion model suggests that proteins contain specific pathways for the transfer of energy from water collisions to catalytic sites. These pathways represent novel targets for drug design. Network analysis methods allow for the consideration of groups of connected protein structures that facilitate energy transfer to find important “hub” components that manage how energy moves through the protein. Sethi et al. [69] demonstrated that targeting such hubs with small molecules may effectively disrupt protein functions.

Compounds that change how different parts of a protein vibrate can interfere with the transfer of energy from water collision areas to the active sites. Leitner [16] suggested that such an approach could lead to a new class of enzyme inhibitors. By focusing on areas that enable the transfer of energy between allosteric and active sites, drugs that change how proteins work without competing with the substances they act on can be designed. Kornev and Taylor [70] found critical structural features in kinases that facilitate energy transfer, which can be targeted by specific inhibitors.

The dynamic energy conversion model suggests that proteins temporarily take on high-energy shapes due to random movement, which is important for their functioning. Drugs targeting these states offer unique advantages; for example, compounds that recognize the shape of high-energy protein forms can attach to them in such a state, keeping them stable and preventing the protein from finishing its catalytic process. Schramm [71] demonstrated that transition state analogs can be exceptionally potent enzyme inhibitors.

Drugs that bind to and stabilize specific conformational states can trap proteins in nonfunctional conformations. Tummino and Copeland [72] showed that such conformational trapping can lead to inhibitors with exceptionally long residence times. Researchers can develop drugs with unique selectivity profiles by designing compounds that selectively bind to proteins in specific energy states (e.g., high- vs. low-energy states). Lu and Wang [73] indicated that the varying movement patterns of similar proteins could be utilized in the creation of targeted inhibitors.

The recognition that substrate binding itself is a dynamic process influenced by Brownian motion suggests novel approaches for drug design. Compounds that disrupt the ways in which substrates move into active sites can successfully stop enzyme activities without having to compete with the substrate directly. Bui et al. [74] described such binding pathways in P450 enzymes and showed they could be considered as targets for the development of novel drugs.

Many enzymes contain flexible elements that act as gates, controlling access to active sites. Drugs that “lock” these gates in closed conformations can effectively inhibit enzyme functions. Kokh et al. [75] identified such gating mechanisms in numerous drug targets and showed how they could be exploited for drug design. Compounds that interfere with the movement of water molecules at protein binding sites can also affect the binding of substrates and the enzyme’s catalytic functioning. Breiten et al. [76] demonstrated that such water-mediated effects can significantly impact the binding affinity and selectivity of proteins.

These new strategies for drug design change the way that we think about it, shifting from the old lock-and-key model to methods that consider how proteins move and change shape. Through targeting proteins as dynamic energy converters rather than static templates, these approaches hold promise for the development of more effective and selective therapeutics.

### 8.2. Rational Design of Enzymes Based on Energy Conversion Principles

The dynamic energy conversion model informs drug design and provides new principles for enzyme engineering. Through explicitly considering how proteins harness Brownian motion to drive catalysis, researchers can design more efficient and versatile biocatalysts for various applications.

Engineering enzymes to more efficiently capture and utilize energy from Brownian motion can enhance their catalytic performance. By modifying α-helices and β-sheets to optimize their mechanical properties, engineers can enhance their ability to absorb and transmit energy derived from water collisions. Huang et al. [77] demonstrated that altering the flexibility of surface-exposed helices could significantly impact the activity of enzymes.

Creating or enhancing pathways for the transfer of energy from surface-exposed regions to catalytic sites can improve catalytic efficiency. Reynolds et al. [78] found that adding mutations to create connected groups of residues can improve communication over long distances and improve the enzyme’s functioning. Adjusting the vibrational properties of enzymes to match specific reaction coordinates can enhance their catalytic efficiency. Hay et al. [79] showed that changing the vibrations of enzymes is essential for hydrogen tunneling reactions, allowing for tailored designs to improve how well they work as catalysts.

Transplanting dynamic elements from one enzyme to another can introduce new functional properties. Dodani et al. [80] found that moving dynamic parts from one enzyme to another allows them to effectively share certain properties, leading to the creation of new biocatalysts.

The right balance of rigidity and flexibility is crucial for enzyme function, and engineering this balance can yield improved biocatalysts. Through selectively introducing flexibility or rigidity in specific regions, engineers can control how enzymes respond to Brownian motion. Pabis et al. [81] demonstrated that such targeted flexibility modifications could enhance the catalytic efficiency and alter the substrate specificity of enzymes.

Changing the hinge areas that manage significant movements in enzymes can alter how they react to Brownian forces and affect their ability to catalyze reactions. Kurkcuoglu et al. [82] identified conserved hinge regions in enzyme families and showed how their modification could alter the enzymes’ dynamics and functions. The transfer of energy through protein structures can be controlled by engineering loops that connect secondary structural elements. Yu and Dalby [83] showed that loop engineering could significantly impact the activity and stability of enzymes by modifying their dynamic properties.

Balancing stability and flexibility is crucial for enzyme functions, as excessive rigidity can impair the dynamic properties which are necessary for catalysis. Tokuriki and Tawfik [84] demonstrated how this balance could be engineered to create enzymes with improved catalytic properties while maintaining sufficient stability.

Understanding proteins as dynamic energy converters enables the design of entirely new catalytic mechanisms. Creating enzymes that leverage the movement of proteins to manipulate the behaviors of substrates can lead to new ways of speeding up chemical reactions. Otten et al. [85] demonstrated that such a strain-based approach could be used to design enzymes for challenging responses.

Designing enzymes with dynamic elements that position cofactors optimally during different stages of catalysis can enhance the catalytic efficiency. Campbell et al. [86] showed how engineering the dynamic positioning of redox cofactors could improve electron transfer reactions. Developing enzymes that leverage changes in their shape to facilitate reactions, instead of relying on heat-dependent energy, can lead to new ways of speeding up chemical processes. Kamerlin and Warshel [87] showed that the randomness in the behavior of enzymes has an important effect on how they work and proposed ways to enhance this randomness.

Developing active sites that can change shape to carry out different reactions can lead to more effective multifunctional enzymes. Nestl et al. [88] demonstrated the feasibility of engineering such multifunctional catalysts through manipulating protein dynamics.

Researchers have developed several computational approaches for the design of enzymes based on dynamic principles. Tools such as Rosetta, with dynamic modules, can incorporate protein motions into the design process. Ollikainen et al. [89] demonstrated that such dynamic-aware design algorithms could help to create enzymes with improved catalytic properties.

Methods for designing proteins with specific energy landscapes can yield enzymes with customized dynamic properties. Li et al. [90] showed how energy landscape engineering could be used to create enzymes with precise control over conformational dynamics. Tools that enable the prediction of how mutations affect the energy conversion efficiency of enzymes can guide their rational design. Romero-Rivera et al. [91] developed methods for predicting the catalytic power of designed enzymes based on their dynamic properties.

Approaches that design against ensembles of conformational states, rather than single structures, can yield more robust enzymes. Friedland and Kortemme [92] demonstrated the advantages of such ensemble-based approaches for enzyme design.

These approaches to rational enzyme design based on energy conversion principles represent a significant advance over traditional design methods, which have focused primarily on static active site geometries. Considering that enzymes leverage Brownian motion-derived energy to speed up chemical reactions, these methods could lead to the development of better and more adaptable biocatalysts enabling many different uses.

### 8.3. Machine Learning Approaches to Predict Dynamic Catalytic States

Beyond AlphaFold, a broader array of machine learning (ML) approaches has been developed to predict and analyze the dynamic states of proteins relevant to catalysis. These methods leverage various data sources and algorithms to capture the complex relationships between protein sequences, structures, and dynamics.

Several supervised learning approaches have been developed to predict dynamic properties directly from protein sequences or structures. ML models trained on experimental B-factors, NMR order parameters, or MD simulation data can predict the flexibility of protein regions directly, based on their sequence or structure. Li et al. [93] demonstrated that deep learning models can accurately predict protein flexibility profiles, providing insights into those regions which likely participate in dynamic energy conversion.

Models trained on pairs of protein structures representing different conformational states can learn to predict potential conformational transitions. Verma et al. [94] showed that graph neural networks can successfully predict how proteins change shape, helping us to understand how they might react to random movements. Specialized machine learning models have been created to predict how the active sites of enzymes change when substrates bind to them and how these conformational changes help in chemical reactions. Amaro et al. [95] demonstrated that such models can identify cryptic binding sites that only become accessible through protein dynamics.

ML approaches can help to predict the transition states and intermediate conformations that proteins adopt during catalysis. Wang et al. [96] showed that variational autoencoders can effectively model the conformational pathways of enzymes, revealing how they navigate energy landscapes during catalysis.

Unsupervised learning approaches have proven valuable for discovering patterns in protein dynamics without relying on labeled training data. Techniques such as PCA, t-SNE, and autoencoders have been used to determine the main ways that proteins change shape, revealing the key paths by which Brownian motion influences protein behaviors. Naritomi and Fuchigami [97] demonstrated that such approaches can efficiently capture the essential dynamics of proteins from simulation data.

Unsupervised clustering methods can determine the different shapes that proteins take on while moving, indicating the specific states they adopt to perform their functions with the help of Brownian motion. Shukla et al. [98] showed that Markov state models created from grouped MD simulations can effectively represent how proteins change shape over time. Unsupervised learning on multiple sequence alignment data can reveal patterns of related mutations that represent how different parts of the protein interact. Morcos et al. [99] demonstrated that such an approach can reveal networks of residues involved in energy transfer and conformational changes.

Methods for analyzing time-series data can extract patterns from protein trajectory data, revealing how proteins respond to and utilize Brownian forces over time. Schwantes and Pande [100] showed that time-lagged independent component analysis can successfully reveal slow movements in proteins that are important for their functioning.

Recent advances in deep learning have enabled more integrated approaches for the modeling of protein dynamics. Graph neural networks represent proteins as graphs, with nodes corresponding to residues or atoms and edges representing interactions. This representation naturally captures the network-like properties of proteins, especially in terms of facilitating the transfer of energy derived from water collisions to catalytic sites. Ingraham et al. [101] demonstrated that graph neural networks can be used to effectively model conformational changes in proteins.

Recurrent neural networks, such as LSTMs or GRUs, enable the tracking of proteins over time, showing how they move through different conformations as time goes on. Tang et al. [102] showed that recurrent architectures can predict how proteins behave over time when affected by Brownian motion. Generative models such as variational autoencoders and generative adversarial networks can learn to create the various shapes that proteins can take, reflecting the different states that proteins adopt due to Brownian motion. Lamim Ribeiro and Tiwary [103] demonstrated that such models can help researchers to efficiently explore the conformational landscape of proteins.

Physics-informed neural networks use the rules of physics in their design, ensuring that their predictions follow the fundamental laws determining the behaviors of proteins. Wang et al. [104] showed that physics-informed models can accurately predict protein dynamics while maintaining physical realism.

The most powerful computational frameworks for studying protein dynamics and catalysis often integrate multiple approaches. Machine learning models can guide MD or BD simulations to efficiently explore relevant regions of conformational space, revealing how proteins utilize Brownian motion to function. Noé et al. [105] demonstrated that such integrated approaches can significantly accelerate the exploration of protein conformational landscapes.

To study catalytic reactions, approaches that combine quantum mechanical calculations for the active site with classical or ML models for the surrounding protein context offer comprehensive perspectives on how protein dynamics influence chemical transformations. Mulholland and colleagues [106] showed that such hybrid approaches allow for the accurate modeling of enzyme catalysis while accounting for protein dynamics. Frameworks integrating information across multiple scales—from quantum effects to macroscopic properties—provide the most complete picture of how proteins harness Brownian motion for catalysis. In this line, Amaro et al. [95] demonstrated that multiscale approaches can connect atomic-level dynamics to macroscopic enzymatic functions.

Computational methods that generate and analyze ensembles of protein structures—rather than single static models—provide the most accurate representation of proteins as dynamic entities constantly responding to Brownian forces. Bottaro and Lindorff-Larsen [107] showed that ensemble-based approaches can accurately capture the dynamic properties of proteins that are observed experimentally.

These computational approaches collectively provide a powerful toolbox for studying how proteins harness Brownian motion for catalysis. Through combining information derived from molecular dynamics simulations, Brownian dynamics models, AlphaFold predictions, and machine learning techniques, researchers can create detailed models of proteins that act as dynamic energy converters instead of just fixed structures.

## 9. Future Directions for Dynamic-Structure-Based Drug Discovery

As our understanding of proteins as dynamic energy converters continues to evolve, several promising directions are emerging for the future of drug discovery and enzyme engineering.

The most powerful future approaches will likely combine experimental and computational methods. New experimental methods such as time-resolved crystallography, cryo-EM, single-molecule FRET, and computer modeling will allow us to visualize how proteins move during chemical reactions or when they bind to drugs. Nogly et al. [108] demonstrated the power of such integrated approaches for visualizing transient conformational states.

Creating techniques that directly observe how proteins move, instead of how they bind or work, will help to find compounds that specifically change these movements. Gooljarsingh et al. [109] pioneered such dynamics-focused screening approaches. Using cycles of computer simulations and lab tests will allow for the rapid improvement of drugs or enzymes based on how they change over time. Rinaldi et al. [110] demonstrated the effectiveness of such an iterative approach for the engineering of enzyme dynamics.

ML models trained on datasets that explicitly include information about protein dynamics are expected to enable more accurate drug binding or enzyme activity predictions. Cyphers et al. [111] discussed how such dynamics-aware ML models could improve virtual screening success rates.

Future approaches may increasingly address the dynamic properties of complex biomolecular systems. Focusing on how protein complexes come together and break apart—which often involves significant conformational changes caused by random movement—is an exciting new area for exploration. Shen et al. [112] identified potential drug targets based on protein complex dynamics.

Focusing on the creation, characteristics, or roles of liquid–liquid phase-separated biomolecular condensates—which are naturally changing structures—is expected to provide new treatment options. Klein et al. [113] identified small molecules that modulate condensate dynamics with potential therapeutic applications. Creating methods that focus on the special movement characteristics of membrane proteins, which work in a mixed-fat environment, may increase the number of potential drug targets. Lee et al. [114] revealed the importance of dynamic properties for membrane protein function and drug binding.

Targeting the dynamic interactions between host and pathogen proteins could yield novel anti-infective strategies. Verteramo et al. [115] demonstrated the potential of targeting dynamic aspects of virus–host protein interactions.

Understanding individual variations in protein dynamics opens possibilities for personalized medicine, and methods for predicting how disease-associated mutations affect protein dynamics could guide personalized treatment decisions. Ponzoni and Bahar [116] demonstrated that many disease mutations alter the dynamics of proteins rather than their stability.

Identifying biomarkers based on altered protein dynamics rather than expression levels or static structures could improve approaches for patient stratification. In this context, Zimmermann et al. [117] identified potential dynamic biomarkers in cancer patients. Choosing drugs based on their ability to normalize altered dynamics in a patient’s specific variant of a target protein could improve treatment outcomes. Vatansever et al. [118] proposed approaches for such dynamics-based drug selection.

Understanding how population-specific genetic variations affect protein dynamics could help to explain differential drug responses across populations. Kumar et al. [119] identified population-specific effects on protein dynamics, which has implications in terms of drug responses.

Several emerging technologies hold promise for advancing dynamic-structure-based approaches. Quantum computing approaches could enable the simulation of protein dynamics at unprecedented scales and accuracies. Outeiral et al. [120] outlined potential applications of quantum computing in protein dynamics modeling.

Advanced AI systems specifically trained to design molecules that modulate protein dynamics could transform drug discovery practices. Popova et al. [121] demonstrated the potential of AI for designing molecules with specific effects on target proteins. Delivery systems that respond to the dynamic states of proteins could enable targeted drug release. Lu et al. [122] developed proof-of-concept systems for such dynamic-responsive delivery.

Tools that track how proteins change in living cells could help to discover drugs that affect these changes in real-life situations. Perkins et al. [123] developed FRET-based sensors for the monitoring of protein conformational changes in vivo.

These future directions highlight the transformative potential of the dynamic energy conversion model in the context of drug discovery and enzyme engineering. By shifting from fixed structural methods to focusing on how proteins harness Brownian motion to function, researchers can create better drugs and biocatalysts that take advantage of the dynamically changing properties of proteins.

## 10. Case Studies of Enzymes Exhibiting Deformation-Dependent Catalysis

**Figure 3:**
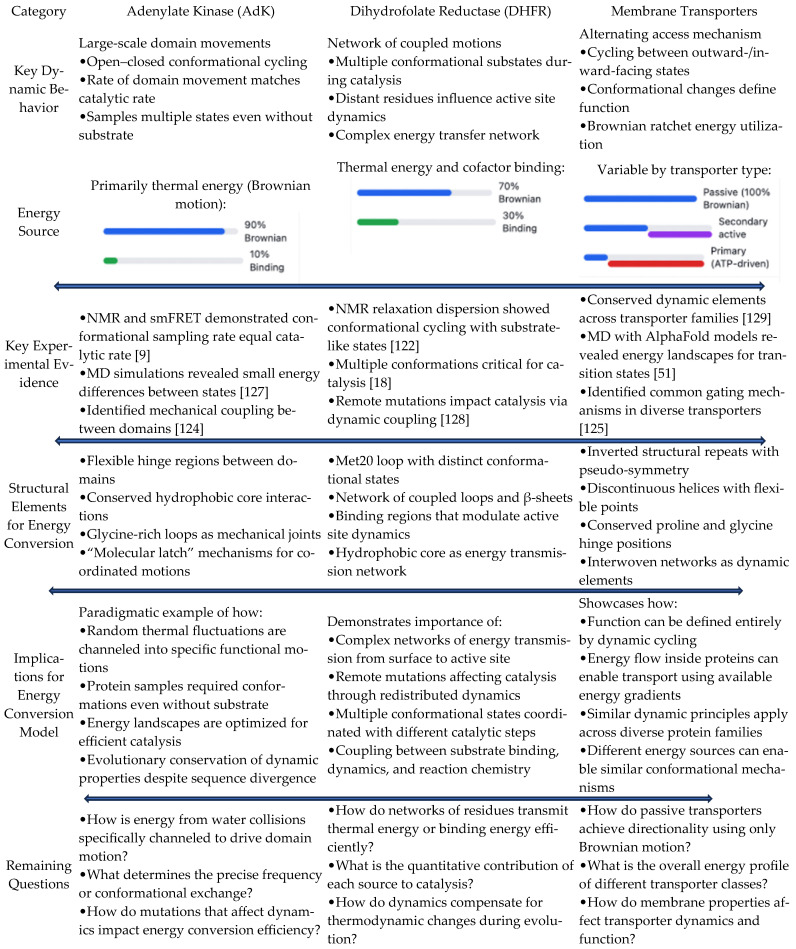
Case studies: proteins as dynamic energy converters [9,18,51,122,124,125,127,128,129].

Many well-researched enzyme systems show clear examples of deformation-dependent catalysis, demonstrating how changes in their shape help them to perform different types of reactions and adopt certain protein structures. Figure 3 summarizes key recent studies in this context.

Adenylate kinase (AdK) helps to transfer a phosphate group back and forth between AMP and ATP to create two ADP molecules. This enzyme serves as a model system for studying the relationship between protein dynamics and catalysis. Henzler-Wildman et al. [9], using NMR and single-molecule FRET, showed that AdK moves significantly during its functioning, switching between “open” and “closed” shapes [124,125,126,127].

The dynamics of AdK exemplify several principles of deformation-dependent catalysis. The enzyme switches between open and closed shapes even when there is no substrate, demonstrating that these conformational changes are a natural part of the protein and are not just caused by substrate binding. The rate of conformational switching closely matches the catalytic rate, suggesting that domain movements are rate-limiting for the overall reaction. The movements of different parts of the enzyme help to position faraway catalytic residues correctly, making it easier to perform phosphate group transfer.

Through computational studies, Müller et al. [11] suggested that AdK uses an “energetic counterweight”, namely, the energy needed to bind the substrate is offset by the energy released when the domains close. This concept aligns with the dynamic energy conversion model, suggesting that the potential energy stored in deformed domains contributes directly to the catalytic process.

Dihydrofolate reductase (DHFR) converts dihydrofolate into tetrahydrofolate using NADPH as a helper molecule. Benkovic and Hammes-Schiffer [3] showed that DHFR performs a complicated series of movements during its catalytic process, where changes in areas far from the active site can affect the efficiency of the process.

Key findings from DHFR studies reveal that changes in the flexibility of the protein—even if they do not directly change the active site—can greatly affect how fast the enzyme functions, highlighting the importance of how different parts of the protein move together. The enzyme cycles through multiple conformational states during catalysis, with each state optimized for a different step in the reaction. Groups of hydrogen bonds and van der Waals interactions help to move energy and information throughout the protein, connecting distant parts of the protein to the active site.

These findings back the idea that DHFR acts like a flexible energy converter, with conformational changes helping it to perform its catalytic role.

F1-ATPase—namely, the catalytic portion of ATP synthase—provides perhaps the clearest example of deformation-dependent catalysis. This rotary motor enzyme uses the energy from proton gradients to drive conformational changes that enable the synthesis of ATP from ADP and phosphate.

Research on F1-ATPase has shown that the enzyme presents significant rotational movements while it works, with each 120° turn linked to the making or breaking of one ATP molecule. The changes in the enzyme’s shape affect how well it can grab onto the starting materials and products, helping to push the reaction along. The energy from the proton gradient is transformed into physical pressure within the protein, which facilitates the synthesis of ATP even when it is not energetically easy.

While F1-ATPase derives its energy from a proton gradient instead of random movement, it exemplifies how conformational changes in proteins can speed up chemical reactions, highlighting the idea that proteins act as active energy converters.

The Natronobacterium gregoryi Argonaute (NgAgo) case represents an intriguing example of how the dynamic energy conversion model can offer novel explanations for controversial experimental findings. In 2016, Han and colleagues [130] reported that NgAgo possessed DNA-guided DNA endonuclease activity, potentially offering an alternative to CRISPR-Cas9 for genome editing. However, multiple laboratories failed to reproduce these results, leading to the retraction of the original paper and considerable controversy in the field.

Han and colleagues [130] stated that NgAgo—using a special type of single-stranded DNA (ssDNA)—could cut double-stranded DNA (dsDNA) at specific locations. This activity would have represented a novel function for Argonaute proteins, which are primarily known for their role in RNA interference pathways. The report generated excitement due to potential advantages over CRISPR-Cas9 for specific applications.

However, numerous laboratories worldwide reported an inability to reproduce the DNA cleavage activity despite confirming that NgAgo could bind both guide and target DNA. This discrepancy led to intense debate regarding the validity of the original findings, with suggestions ranging from technical difficulties to laboratory-specific conditions that might enable the reported activity.

One potential explanation, which is consistent with the dynamic energy conversion model, is that while NgAgo may possess the structural capability to bind guide DNA and target DNA (which most laboratories confirmed), the potential energy generated due to NgAgo’s structural deformation under certain experimental conditions might only occasionally reach the threshold required for DNA cleavage. This idea implies that the energy needed for NgAgo to function as a catalyst is very close to that which can be produced when proteins move in standard lab settings.

This concept means that factors such as temperature, the makeup of the solution, or how crowded the molecules are can significantly affect whether NgAgo has enough energy due to its conformation to cleave DNA. This variability highlights the importance of optimizing experimental conditions to ensure that NgAgo can effectively perform its catalytic function. Understanding these factors can improve gene editing applications and molecular biological research. This hypothesis suggests that Han et al. may have inadvertently created conditions where NgAgo’s deformation energy occasionally exceeded the threshold for DNA cleavage, while other laboratories operated under conditions where this threshold was not reached.

Several observations support this hypothesis. Some reports have suggested that temperature significantly affects the activity of NgAgo, which aligns with the expectation that higher temperatures would increase the frequency and magnitude of deformation events, potentially enabling DNA cleavage. Variations in buffer composition between laboratories could affect protein dynamics and energy storage capacity, potentially explaining the differences in observed activity. Even in the original report, the efficiency of NgAgo-mediated DNA cleavage was relatively low, consistent with the idea that the energy threshold for cleavage was only occasionally reached.

It is important to emphasize that this interpretation remains a hypothesis requiring further experimental verification. However, it illustrates how the dynamic energy conversion model can provide new frameworks for understanding controversial or inconsistent experimental results in the field of enzyme catalysis.

This case highlights several important implications of the dynamic energy conversion model for enzyme research. The concept that enzymes require a specific threshold of potential energy to catalyze reactions suggests that borderline cases might exist where slight changes in conditions can dramatically affect activity. Understanding the factors that influence the deformation energy of proteins could enable the deliberate engineering of enzymes with enhanced catalytic abilities or more consistent performance across different conditions.

Thus, the NgAgo case is an illustrative example of how the dynamic energy conversion model can provide new perspectives on enzyme functions, potentially offering explanations for experimental inconsistencies that traditional static models struggle to accommodate.

Membrane transport proteins represent a fascinating class of enzymes where conformational dynamics are not merely coupled to function but essentially constitute the function itself. These proteins facilitate the movement of ions, metabolites, and other molecules across cellular membranes by cycling through different conformational states that alternately expose their binding sites to opposite sides of the membrane. Their function thus provides an excellent case study regarding the principles of dynamic energy conversion in proteins.

Most transporters operate via an “alternating access” mechanism, where the protein cycles between at least three major conformations: an “outward-facing” state, with the binding site accessible from one side of the membrane; an “occluded” state, with the binding site inaccessible from either side; and an “inward-facing” state, with the binding site accessible from the opposite side. This conformational cycle effectively translocates bound substrates across the membrane barrier. Importantly, these conformational changes require energy, which can come from various sources depending on the transporter.

Different classes of transporters utilize different energy sources to drive their conformational cycles. Primary active transporters (e.g., P-type ATPases, ABC transporters) directly couple ATP hydrolysis to conformational changes, converting chemical energy into mechanical work. Secondary active transporters (e.g., symporters, antiporters) utilize electrochemical gradients of ions (typically Na^+^ or H^+^) to drive substrate transport. Facilitated diffusion transporters (e.g., GLUT family) harness the concentration gradient of the transported substrate itself, with Brownian motion providing the energy for conformational changes.

The dynamic energy conversion model is particularly relevant for understanding how transporters in the third category function, as they rely entirely on thermal energy from Brownian motion to drive their conformational cycles.

The oxalate transporter (OxlT) from Oxalobacter formigenes provides an excellent example of how Brownian motion drives transport functions. This protein exchanges oxalate for formate across the bacterial membrane, playing a crucial role in oxalate metabolism and potentially preventing kidney stone formation in humans.

Recent structural and computational studies of OxlT have provided detailed insights into its conformational dynamics. Crystal structures have captured OxlT in outward-open and occluded states, but the inward-open conformation had remained elusive until recently. Ohnuki and Okazaki [51] combined accelerated molecular dynamics simulations with AlphaFold predictions to identify the missing inward-open conformation, demonstrating that this state preferentially binds formate over oxalate.

The simulations revealed energy barriers between the different conformational states that are low enough to be overcome by thermal energy alone, explaining how Brownian motion can drive the complete transport cycle. The analysis also identified networks of residues that stabilize different conformational states and mediate transitions between them, effectively functioning as energy storage and conversion elements.

These findings strongly support the view of OxlT as a dynamic energy converter that harnesses Brownian motion to drive substrate transport, with specific structural elements that evolved to store and release energy during the transport cycle.

Membrane transporters typically contain multiple α-helices arranged in complex patterns, with these secondary structural elements playing crucial roles in energy conversion. Many transporters contain relatively rigid transmembrane helices that move as semi-rigid bodies during conformational changes, efficiently transferring energy throughout the structure. Specific regions between helices serve as flexible hinges that allow for large-scale conformational changes while maintaining structural integrity.

Features such as proline kinks and glycine residues create specific points of flexibility in transmembrane helices, allowing them to bend or break during conformational transitions. Internal water molecules often form dynamic networks that mediate interactions between helices and participate in the transfer of energy throughout the structure.

Drew and Boudker [129] demonstrated that these structural features are often conserved across transporter families, suggesting evolutionary optimization for efficient energy conversion. This aligns with the concept proposed by Cao and Ding [7], who stated that α-helices and β-sheets serve as key elements for harnessing energy derived from Brownian motion.

Membrane transporters provide important insights supporting and extending the dynamic energy conversion model. Transporters demonstrate how proteins can function through energy absorption, storage, and release, with each stage of the cycle involving a specific functional role. The existence of multiple stable conformational states with relatively small energy differences between them enables the efficient conversion and utilization of energy.

The binding of substrates often alters the energy landscape of transporters, illustrating how ligand interactions can modulate protein dynamics and energy conversion. Comparative studies across transporter families have revealed the conservation of dynamic properties despite sequence divergence, suggesting evolutionary pressure selecting for efficient energy conversion.

The study of membrane transporters thus provides strong support for the dynamic energy conversion model while highlighting how proteins can evolve to harness Brownian motion for diverse functional purposes beyond traditional catalysis.

## 11. Allosteric Enzymes: Integration of Dynamics and Regulation

Allosteric enzymes represent a class of proteins for which conformational dynamics play a dual role: enabling catalysis and mediating regulation through communication between distinct binding sites. These enzymes provide compelling examples of how proteins can function as integrated dynamic systems that harness Brownian motion for catalytic and regulatory purposes.

Allostery is fundamentally a dynamic phenomenon involving communication between spatially separated sites through conformational changes and altered dynamics. Several models have been developed to describe this process; for example, the Monod–Wyman–Changeux (MWC) and Koshland–Némethy–Filmer (KNF) models describe allostery in concerted or sequential conformational changes between distinct states.

More recent models emphasize changes in protein dynamics rather than just structure, with allosteric effects propagating through altered vibrational modes and entropy. Population-shift models describe allostery as a redistribution of pre-existing conformational states rather than creating new conformations, emphasizing the role of Brownian motion in the adoption of different protein configurations.

The dynamic energy conversion model provides a physical basis for understanding these phenomena. It suggests that allosteric effectors alter how proteins store and transmit energy derived from Brownian motion, thereby modulating their catalytic properties.

DHFR, which catalyzes the reduction of dihydrofolate to tetrahydrofolate using NADPH as a cofactor, serves as a model system for understanding the integrated effects of dynamics and allostery on enzyme functions.

Key findings from DHFR studies include identifying a network of residues that exhibit coordinated motions during catalysis, connecting the active site to distant regions of the protein. An NMR study conducted by Wright and colleagues [131] revealed that DHFR exhibits dynamics across multiple timescales, from local fluctuations at the picosecond scale to conformational exchanges at the millisecond scale, with these different motions playing distinct roles in its catalytic functioning.

Cameron and Benkovic [128] demonstrated that mutations far from the active site could significantly impact catalytic activity by altering the dynamic network of the enzyme. A comparative study performed by Bhabha et al. [18] revealed that DHFR enzymes from different species exhibit distinct dynamic properties despite their structural similarity, suggesting evolutionary tuning of dynamics for specific functional requirements.

These observations support the view of DHFR as a dynamic energy converter, where networks of coupled residues transmit energy derived from Brownian motion throughout the structure, with allosteric effects modulating these energy pathways to regulate certain functions.

Computational studies have provided detailed insights into the physical basis of allosteric communication through protein dynamics. Sethi et al. [69] performed network analysis of MD simulations to identify communities of residues that move together, revealing how energy and information propagate through protein structures.

Bahar and colleagues [37], using elastic network models, demonstrated that allosteric effects often propagate through low-frequency collective motions that efficiently transmit energy across long distances. Nussinov and Tsai [132] conceptualized allostery in terms of remodeling of the energy landscape, where effectors alter the population of different conformational states by modifying the energy barriers between them.

Shannon et al. [133] applied information theory to quantify how effectively different protein regions communicate, revealing the pathways through which allosteric signals propagate.

These computational approaches have enabled the identification of physical mechanisms demonstrating how proteins can efficiently transmit the effects of binding events over long distances, supporting the concept that proteins are integrated dynamic systems that process and utilize energy derived from Brownian motion.

The study of allosteric enzymes extends the dynamic energy conversion model in several important ways. Allosteric effectors can alter the capacity of proteins to store potential energy derived from Brownian motion, thereby modulating their catalytic efficiency. The binding of allosteric effectors can redirect the flow of energy through protein structures, changing which regions receive sufficient energy for conformational changes relevant to catalysis.

Allosteric effects often involve changes in energy barriers between conformational states, effectively controlling how proteins navigate their energy landscapes in response to Brownian motion. Many allosteric systems integrate energy from multiple sources—including Brownian motion, ligand binding, and covalent modifications—demonstrating the sophisticated energy management capabilities of these proteins.

These principles are evident in various allosteric enzymes, from hemoglobin’s cooperative oxygen binding to the regulation of protein kinases by phosphorylation events. In each case, the protein functions as an integrated dynamic system that processes and utilizes energy from various sources; in particular, Brownian motion provides a constant input that drives the adoption of different functional states.

The study of allosteric enzymes thus highlights the sophistication of proteins as dynamic energy converters which are capable of harnessing Brownian motion for catalysis and integrating multiple inputs to regulate their activity in response to cellular needs in a precise manner. This perspective extends the dynamic energy conversion model from a mechanism of catalysis to a broader framework for understanding the functions and regulation of proteins in complex biological systems.

## 12. Evolutionary Perspectives

### 12.1. Selection Pressures on Protein Dynamics and Catalytic Efficiency

The dynamic energy conversion model introduces a new perspective regarding the evolutionary forces that have shaped protein structures and functions. If proteins function as dynamic energy converters that harness Brownian motion for catalysis, then evolutionary selection would be expected to optimize their capacity to absorb, store, and utilize energy from their environment. This perspective reveals several distinct selection pressures that may have driven the evolution of proteins.

Proteins must efficiently absorb energy from water collisions to drive catalysis, suggesting selection pressure for structures that effectively capture this energy. Larger surface areas increase the frequency of water collisions but must be balanced against the potential dissipation of energy and stability of the protein. Grishin [134] demonstrated that proteins across diverse families show evidence of evolutionary optimization in terms of their surface-to-volume ratios.

The flexibility of surface regions affects how effectively they can absorb energy from collisions. Fuglebakk et al. [135] showed that enzyme surface residues exhibit evolutionary patterns consistent with selection for specific flexibility profiles. The distribution of α-helices and β-sheets on protein surfaces influences their energy absorption efficiency. Dokholyan and Shakhnovich [136] revealed that the arrangement of secondary structures in natural proteins often follows patterns that optimize mechanical properties.

Some proteins present surface features that may function specifically to absorb energy from the environment; for example, Glykos et al. [137] reported unusually flexible surface loops in certain enzymes, which could serve as energy-capturing “antennae.”

These observations suggest that proteins have evolved surface features optimized explicitly for absorbing energy from Brownian motion, supporting the dynamic energy conversion model for protein functions.

Once energy is absorbed, proteins must efficiently store it as potential energy that can then be utilized for catalysis. The network of interactions within proteins determines their capacity to store potential energy through deformation. Haliloglu and Bahar [138] demonstrated that natural proteins exhibit elastic network properties which are optimized for energy storage and transmission.

Proteins with multiple stable or metastable states separated by modest energy barriers can effectively store and utilize energy derived from Brownian motion. Tsai et al. [139] showed that such metastable states are common in enzymes and appear to be evolutionarily conserved. Specific regions within proteins may have evolved to store strain energy. Lu et al. [140] identified conserved structural motifs in enzymes that appear to be designed to accumulate strain under deformation.

Interfaces between protein domains often store potential energy through electrostatic and van der Waals interactions. Gerstein et al. [141] demonstrated the evolutionary conservation of domain interface properties that are consistent with optimized energy storage.

These energy storage mechanisms appear to be explicitly selected for during the evolution of proteins, suggesting that the ability to store potential energy derived from Brownian motion is an essential determinant of a protein’s fitness.

The link between energy conversion and catalytic activity suggests the existence of specific selection pressures relating to how proteins utilize stored energy. Active sites are often positioned to receive energy efficiently from deformation-prone regions. Tawfik and colleagues [142] revealed evolutionary patterns in active site positioning which are consistent with optimization for the receipt of energy from specific structural elements.

Many enzymes contain structural features that focus energy from multiple regions onto specific catalytic residues. Daily and Gray [143] identified networks of conserved interactions that may serve as enzyme energy conduits. The dynamics of proteins often appear to be specifically coupled to reaction coordinates. Schwartz and colleagues [144] demonstrated that evolutionary conservation patterns in enzymes frequently correlate with dynamical features which are relevant to their catalytic mechanisms.

Evolutionary processes must balance catalytic efficiency (often requiring flexibility) against stability (often requiring rigidity). In particular, Tokuriki and Tawfik [84] revealed how proteins navigate this trade-off through specific structural adaptations.

These observations suggest that natural selection has fine-tuned the dynamic properties of proteins to efficiently convert stored potential energy into catalytic activity, supporting the central thesis of the dynamic energy conversion model.

Adapting proteins to environments characterized by different temperatures provides compelling evidence for the selection of dynamic properties. Proteins from organisms living at different temperatures show consistent differences in flexibility and rigidity. Siddiqui and Cavicchioli [145] revealed that psychrophilic (cold-adapted) enzymes typically show enhanced flexibility, while thermophilic (heat-adapted) enzymes show increased rigidity, consistent with the optimization of dynamic properties with respect to different thermal environments.

Enzymes must maintain their activity (requiring dynamics) and stability within their range of operating temperatures. Åqvist et al. [146] demonstrated specific evolutionary strategies for achieving this balance across temperature ranges. The energy barriers between conformational states appear to be tuned to the thermal energy available in the organism’s environment. Wolf-Watz et al. [147] showed that homologous enzymes from organisms living at different temperatures exhibit corresponding adjustments in their energy landscapes.

Despite sequence divergence, enzymes often preserve specific dynamic properties across different thermal adaptations. Isaksen et al. [148] identified conserved dynamic signatures in enzyme families spanning diverse thermal niches.

These temperature adaptation patterns provide strong evidence that protein dynamics are directly subject to natural selection, supporting the view of proteins as dynamic systems that harness thermal energy for their functioning.

### 12.2. Evolutionary Conservation of Dynamic Elements in Enzyme Families

If protein dynamics play a critical role in catalysis through energy conversion, one would expect the evolutionary conservation of structural elements involved in these dynamic processes. Recent studies combining evolutionary analysis with dynamic characterization have revealed conservation patterns that extend beyond the static structural features traditionally assumed to drive selection.

Contrary to the expectation that functionally important regions would show high sequence conservation and structural rigidity, many enzymes exhibit conserved flexibility patterns. Fraser et al. [19] identified loops with conserved flexibility profiles across enzyme families, suggesting selection for specific dynamic properties rather than just sequences or structures. Despite low sequence conservation, some enzyme families contain intrinsically disordered regions with conserved locations and dynamic properties. Brown et al. [149] demonstrated that these disordered regions often play crucial roles in substrate recognition or product release. Many enzymes show conserved patterns of flexibility gradients across their structures, with specific transitions from rigid to flexible regions. Marsh and Teichmann [150] revealed that these gradients often correlate with functional pathways through the protein. Specific sites within enzymes may present conserved dynamic properties independent of sequence conservation. Kurkcuoglu et al. [82] identified such dynamic hotspots in diverse enzyme families and demonstrated their functional importance. These patterns of conserved flexibility support the dynamic energy conversion model, suggesting that the ability to undergo specific deformations is subject to evolutionary selection.

The networks of residues that transmit energy and information through protein structures show strong evidence of evolutionary conservation. Statistical analysis of multiple sequence alignments often reveals networks of co-evolved residues that form physical pathways through protein structures. Halabi et al. [151] demonstrated that these networks—termed “protein sectors”—often correspond to dynamically coupled regions. Standard mode analysis of homologous enzymes frequently reveals the conservation of low-frequency collective motions despite sequence divergence. Bakan and Bahar [152] showed that these conserved motions often correlate with functionally critical conformational changes. Simulations of the flow of energy through protein structures also suggest the conservation of specific pathways despite sequence variation. Leitner et al. [16] identified conserved energy transfer channels in enzyme families that appear to be optimized for the coupling of surface dynamics with catalytic sites. The pattern of dynamic coupling between distant sites in proteins often shows evolutionary conservation. Henzler-Wildman et al. [9] demonstrated such conserved coupling patterns in the adenylate kinase family, suggesting selection for specific energy transfer properties. These conserved allosteric networks indicate that proteins have evolved to maintain specific dynamic properties that allow for the efficient conversion of Brownian motion into catalytic energy. The relationships between sequences, structures, and dynamics appear to also be subject to specific evolutionary constraints. Mutations that alter dynamic properties often co-occur with compensatory mutations that restore critical dynamic features. Whitley et al. [153] identified patterns of such compensatory mutations in enzyme families, suggesting selection to maintain specific dynamic properties. Despite sequence divergence, many enzyme families show remarkable conservation of dynamic properties, suggesting evolutionary mechanisms that confer robustness to dynamics despite sequence changes. Glembo et al. [154] revealed how proteins achieve this dynamic robustness through distributed interaction networks. Some residues show high conservation despite not being directly involved in catalysis or stability, which is due to their roles in maintaining specific dynamic properties. Tee et al. [155] identified such dynamics-driven conservation patterns in multiple enzyme families.

In some cases, proteins with highly similar structures exhibit different dynamic properties due to subtle sequence variations, suggesting the independent evolution of structure and dynamics. Nashine et al. [156] demonstrated such decoupling in enzyme homologs with distinct catalytic efficiencies. These evolutionary constraints on the sequence–structure–dynamics relationship highlight the importance of dynamic properties in the functions and evolution of proteins, consistent with the dynamic energy conversion model. Laboratory evolution experiments have provided direct evidence for selection with respect to protein dynamics. Directed evolution experiments selecting for increased enzyme activity often yield mutations that alter the dynamic properties of the protein rather than directly affecting the active site. Campbell et al. [86] demonstrated that many activity-enhancing mutations in laboratory-evolved enzymes act by modifying protein dynamics. Experimental evolution studies have revealed complex trade-offs between stability and function, often involving adjustments to dynamic properties. Romero and Arnold [157] showed how proteins navigate these trade-offs during evolutionary optimization. Some neutral mutations revealed by experimental evolution have been shown to affect protein dynamics in advantageous ways under certain conditions. Campell et al. [86] identified such cryptic effects in laboratory-evolved enzymes. The dynamic properties of proteins show different degrees of evolvability, with some dynamic features being more easily modified than others. Tokuriki and Tawfik [84] demonstrated how this differential evolvability shapes evolutionary trajectories. These experimental evolution studies provide direct evidence that protein dynamics are under selection during adaptation, supporting the view that effectively converting Brownian motion into catalytic energy is an essential determinant of fitness for certain proteins.

#### The Origin of α-Helices and β-Sheets as Energy-Harnessing Structures

The dynamic energy conversion model raises intriguing questions about the evolutionary origin of protein secondary structures. If α-helices and β-sheets indeed function as key elements for harnessing energy derived from Brownian motion, their emergence and conservation throughout the process of protein evolution takes on new significance.

Evidence suggests that α-helices and β-sheets may have emerged early in the evolution of proteins. Both α-helices and β-sheets represent low-energy conformations accessible to simple polypeptides, suggesting that they could have emerged spontaneously in prebiotic environments. Guo et al. [158] demonstrated the formation of these structures in prebiotically plausible peptide mixtures. Metal ions, which were present in early Earth environments, can catalyze and stabilize the formation of secondary structures. Behe and Snyder [159] showed that specific metal ions promote helical conformations in simple peptides, potentially facilitating the emergence of structured proteins. Cycles of hydration and dehydration, which are standard in prebiotic settings, can promote the formation and selection of stable secondary structures. Forsythe et al. [160] demonstrated how cycling in this manner could have driven the emergence of structured peptides. Early thermal or chemical stability selection would have also favored peptides adopting regular secondary structures. Gounaris et al. [161] suggested that such selection could have driven the early evolution of α-helices and β-sheets. These observations indicate that the basic secondary structures that enable the absorption of energy derived from Brownian motion could have emerged early in the evolution of proteins, potentially driven by their mechanical properties.

The specific mechanical properties of α-helices and β-sheets suggest that they may have been selected for their energy-harnessing capabilities. Both structures can store potential energy through specific deformation modes: compression/extension and bending for α-helices, shearing and bending for β-sheets. Buehler and Keten [162] characterized these mechanical properties using computational methods, revealing their exceptional energy storage capabilities. The regular hydrogen bonding patterns in both structures allow them to absorb energy from environmental interactions efficiently without structural failure. Rief et al. [14], using single-molecule force spectroscopy, demonstrated the remarkable mechanical resilience of these structures. Both structures exhibit direction-dependent mechanical properties, allowing them to transmit forces preferentially along specific pathways. Dietz and Rief [163] assessed these anisotropic mechanical properties and their potential functional significance. The mechanical properties of α-helices and β-sheets scale favorably with size, allowing larger structures to maintain efficient energy absorption and transmission properties. Keten and Buehler [164] demonstrated how these scaling properties enable the hierarchical assembly of mechanically robust protein structures. These mechanical advantages suggest that the emergence and conservation of secondary structures may have been driven, at least in part, by their capacity to harness energy derived from Brownian motion, supporting the dynamic energy conversion model of protein function.

Secondary structures show evidence of evolutionary diversification to serve different mechanical roles. Different helices (α, 310, π) exhibit distinct mechanical properties, suggesting their evolutionary specialization for different energy-harnessing roles. Baker et al. [165] characterized these differences and their distribution across protein families. Various β-sheet topologies (parallel, antiparallel, mixed) also present different mechanical behaviors, potentially reflecting their adaptation to different functional requirements. Keten et al. [166] mapped these mechanical differences and their potential functional implications. The length distribution of secondary structures in natural proteins often corresponds to mechanical optima for specific functions. Emberly et al. [167] demonstrated that these distributions show evidence of selection for mechanical efficiency. Specific amino acid compositions in secondary structures appear to be selected to fine-tune their mechanical properties. Watanabe et al. [168] revealed patterns of side-chain conservation that are consistent with the optimization of mechanical behavior. These patterns of evolutionary divergence suggest that secondary structures have been refined to serve specific mechanical roles in the context of energy conversion, supporting the view that their energy-harnessing capabilities are functionally important.

The arrangement of secondary structures into super-secondary motifs may further enhance their energy-harnessing capabilities. Common super-secondary structures such as the βαβ motif show evidence of optimization for energy transfer between elements. Glykos et al. [137] demonstrated how these arrangements facilitate energetic coupling between different structural components. Arrangements of multiple helices into bundles form systems with emergent mechanical properties beyond those of individual helices. Yu et al. [169] characterized these emergent properties and their potential roles in energy management. Closed β-barrel structures exhibit unique mechanical properties that enable the efficient absorption and transmission of energy. Perilla et al. [170] revealed how these properties contribute to the function of barrel-containing proteins. Similar structural arrangements have evolved independently multiple times, suggesting selection for specific mechanical properties. Lupas et al. [171] documented the convergent evolution of such mechanically advantageous structural motifs. These super-secondary arrangements suggest that the evolution of proteins has progressed beyond simple secondary structures to the creation of more sophisticated energy-harnessing systems, consistent with the dynamic energy conversion model.

### 12.3. Co-Evolution of Protein Dynamics and Cellular Environments

The dynamic energy conversion model suggests the existence of an intimate relationship between protein dynamics and cellular environments, as the efficiency of energy conversion from Brownian motion depends on the properties of the surrounding medium. This relationship implies co-evolutionary processes that have shaped protein dynamics and cellular conditions throughout evolutionary history.

The efficiency of transferring energy derived from water collisions depends on the viscosity of the cellular environment. Proteins from organisms living in environments with different viscosities reveal corresponding adaptations in their dynamic properties; for example, Fields [172] demonstrated that proteins from deep-sea organisms adapt to high-pressure, high-viscosity environments through specific structural and dynamic features.

The crowded intracellular environment significantly alters the dynamics of Brownian motion, and proteins show evidence of adaptation to these conditions. Zhou et al. [173] revealed how proteins have evolved to function optimally under crowded conditions, which differ from dilute in vitro settings. Proteins that function in or near membranes experience distinct dynamic environments and thus show specific adaptations in their energy-harnessing mechanisms. Baaden and Marrink [174] characterized these adaptations in membrane-associated proteins.

Different organisms maintain distinct cytoplasmic compositions, potentially creating selective pressure for specific dynamic adaptations. Record et al. [175] documented how the cytoplasmic composition varies across organisms and influences protein function.

These adaptations to environmental viscosity suggest co-evolution between protein dynamics and cellular conditions, with proteins optimizing their energy-harnessing capabilities with respect to specific environments.

Temperature affects protein dynamics and the energy that can be derived from Brownian motion, creating complex selective pressures. Proteins from organisms adapted to different temperature ranges show corresponding adjustments in their energy landscapes, suggesting the co-evolution of such dynamics with thermal environments. Åqvist et al. [146] demonstrated how these adjustments maintain similar practical energy barriers despite different thermal energies.

Homologous enzymes from organisms adapted to different temperatures often show similar catalytic rates at their respective physiological temperatures, suggesting compensatory mechanisms to maintain efficient energy conversion. Somero [176] documented this “corresponding states” type of adaptation across temperature ranges. The range of dynamics accessible to proteins appears to be tuned to the temperature fluctuations they experience in their native environments. Fields [172] showed how proteins from thermally stable environments exhibit different dynamic adaptation strategies when compared to those from fluctuating environments.

Some organisms employ metabolites that stabilize protein dynamics against temperature fluctuations, suggesting the co-evolution of cellular chemistry and protein dynamics. Yancey [177] characterized these compatibility solutes and their effects on protein functions across temperature ranges.

The existence of such temperature adaptation mechanisms highlights the intimate relationship between environmental conditions, the availability of Brownian motion-derived energy, and the dynamic properties of proteins, further supporting the dynamic energy conversion model.

The energy conversion capabilities of proteins appear to have co-evolved with broader metabolic systems. The concentrations of ATP and other energy currencies vary across organisms, potentially driving adaptations in protein dynamics to maintain an appropriate energy balance. Park et al. [178] demonstrated correlations between metabolic strategies and protein dynamic properties across diverse organisms.

The cellular redox environment affects protein dynamics through the modification of disulfide bonds and other redox-sensitive elements. Jones [179] presented evidence for the co-evolution of protein dynamic elements with cellular redox systems. The availability and utilization of different metal ions, which can significantly affect protein dynamics, provide evidence for co-evolutionary adaptation. Dupont et al. [180] documented how the utilization of metals in proteins correlates with their environmental availability and cellular homeostasis mechanisms.

Organisms use different osmolyte systems to maintain cellular water activity, and these osmolytes can significantly affect protein dynamics. Yancey et al. [181] provided evidence for the co-evolution of protein dynamics with organism-specific osmolyte strategies.

These co-evolutionary relationships between protein dynamics and metabolic systems suggest that the energy conversion capabilities of proteins are integrated as part of broader cellular energetic strategies.

The dynamics of pathogen and host proteins show evidence of co-evolutionary arms races. Pathogen proteins often exhibit unusual dynamic properties that enhance their function while evading host recognition. van der Lee et al. [182] demonstrated how viral proteins utilize intrinsic disorder and dynamic conformational diversity to evade immune detection while maintaining functionality.

Host receptors involved in pathogen recognition show evidence of selection for specific dynamic properties that balance their recognition sensitivity with specificity. Moeller et al. [183] revealed how the dynamics of immune receptors have evolved in response to pathogenic pressures. Some pathogen proteins mimic the dynamic properties of host proteins to interfere with their normal function. Elde and Malik [184] documented cases of such dynamic mimicry and discussed its role in host–pathogen interactions.

Proteins from obligate parasites show evidence of adaptation to the specific dynamic environments of their hosts. Kappeli and Xiao [185] characterized such adaptations in parasite proteins and detailed their functional significance.

These examples of dynamic co-evolution in host–pathogen systems highlight the functional importance of protein dynamics and further support the view that the ability to harness energy derived from Brownian motion is under direct selective pressure.

The evolutionary perspectives presented in this section strongly support the dynamic energy conversion model of protein functioning. The evidence for the selection of dynamic properties, conservation of energy-harnessing structural elements, and co-evolution of proteins with their environments all suggest that the ability to convert Brownian motion into catalytic energy is a fundamental aspect of protein functioning that has shaped their evolution throughout history. The ubiquity of α-helices and β-sheets across all domains of life may reflect not just their structural stability but also their exceptional capabilities as energy-harvesting devices that enable the remarkable catalytic efficiency of enzymes.

## 13. Challenges and Future Directions

### 13.1. Methodological Challenges in Studying Transient Protein States

The dynamic energy conversion model proposes that proteins utilize high-energy conformational states driven by Brownian motion to catalyze reactions. However, studying these transient, high-energy states poses significant methodological challenges that must be addressed in order to validate and develop this model entirely.

Many high-energy states exist only briefly, requiring high-speed detection methods. The initial absorption of energy during water collisions occurs at picosecond to nanosecond timescales, which is beyond the reach of many experimental techniques. Recent advances in time-resolved X-ray scattering, such as that discussed by Arnlund et al. [186], have begun to access these timescales; however, broader applications remain challenging.

The redistribution of absorbed energy throughout protein structures occurs at nanosecond to microsecond timescales, representing a gap between the capabilities of spectroscopic methods (typically sub-nanosecond) and structural methods (typically millisecond or slower). New methodologies bridging this “time gap” are thus required.

Although many experimental techniques require the synchronization of molecular events across large populations, Brownian-driven processes are inherently stochastic. The technique developed by Henzler-Wildman and Kern [2] has led to progress in addressing this challenge, involving the careful control of temperature and use of reversible triggering methods.

Methods with high time resolution often provide limited structural information, while high-resolution structural methods typically have poor time resolution. Integrative approaches combining multiple techniques—as demonstrated by van den Bedem and Fraser [187]—represent a promising strategy to overcome this trade-off.

The transient nature of high-energy states makes them particularly difficult to characterize structurally. Crystal packing forces can suppress the high-energy conformations relevant to solution-phase catalysis. Methods such as room temperature and time-resolved crystallography—pioneered by Fraser et al. [19] and Chapman et al. [188]—offer potential solutions in this regard but remain technically demanding.

While NMR allows for the detection of conformational exchange, directly characterizing less-populated high-energy states remains difficult. Recent advances in relaxation dispersion and chemical exchange saturation transfer (CEST) methods, such as those detailed by Kay and colleagues [24], have improved access to these states but still face limitations related to sensitivity.

Single-particle cryo-EM typically averages over multiple conformations, potentially obscuring high-energy states. New classification algorithms, such as that developed by Nakane et al. [20], show promise for separating conformational substates but remain limited in terms of the number of particles and image quality.

Molecular dynamics simulations struggle to capture high-energy states due to the rare nature of these conformations. Enhanced sampling methods, such as that developed by Hamelberg et al. [189], partially address this issue but remain computationally expensive and potentially biased.

Quantifying the energetics of protein dynamics poses unique challenges. Measuring how efficiently proteins convert collision energy into potential energy remains technically challenging. As demonstrated by Zhang et al. [190], new optical trapping methods combined with single-molecule fluorescence offer potential approaches, but such methods have not yet been widely applied.

Determining how energy is distributed throughout protein structures requires site-specific probes with minimal perturbation to the system. Recent advances in genetically encoded optical probes, such as that described by Greenwald et al. [191], provide promising tools but still face challenges in terms of comprehensive coverage.

In living cells, proteins utilize multiple energy sources beyond Brownian motion, including ATP hydrolysis and electrochemical gradients. Experimental designs that selectively manipulate specific energy sources, such as that described by Bustamante and colleagues [28], show promise for addressing this challenge.

Connecting observed dynamic behaviors with underlying energy distributions also remains conceptually and technically challenging. Integrated approaches combining single-molecule experiments with computational modeling, as demonstrated by Deniz and colleagues [192], represent a promising strategy in this regard.

These methodological challenges highlight the need for new experimental and computational approaches which are specifically designed to test the predictions of the dynamic energy conversion model. Progress in these areas will be essential for moving from a qualitative understanding to a quantitative, predictive framework for the assessment of the effects of protein dynamics on catalysis.

### 13.2. Integration of AlphaFold Predictions with Dynamic Functional Models

The revolution in protein structure prediction driven by AI methods—particularly AlphaFold—presents both opportunities and challenges in terms of understanding the dynamics and energy conversion ability of proteins. While AlphaFold has dramatically expanded our structural knowledge of the proteome, integrating these static structural predictions with dynamic functional models requires addressing several key challenges.

AlphaFold typically predicts a single static structure, whereas the dynamic energy conversion model emphasizes the importance of conformational ensembles. Existing AI methods have not been explicitly designed to indicate the diverse conformational states of protein samples under Brownian motion. Pearce and Zhang [193] have recently begun to address this limitation by developing modified versions of AlphaFold that can predict multiple conformational states; however, these approaches remain in early development.

The per-residue confidence scores (pLDDT) provided by AlphaFold often correlate with flexible regions but do not directly quantify dynamic properties. Del Alamo et al. [194] explored the relationship between these confidence metrics and protein dynamics, suggesting potential approaches for extracting dynamic information from static predictions. When generating multiple models, it remains challenging to determine whether the sampled conformations adequately represent the dynamic protein ensemble. In this line, the method developed by Ovchinnikov and colleagues [195] for assessing the completeness of conformational sampling shows promise, but requires further validation.

AlphaFold was trained primarily on crystal structures, which may under-represent the high-energy conformational states that are important for catalysis. Approaches for the reweighting of training data or incorporating solution-phase structural information, such as that proposed by Jumper et al. [196], could potentially address this bias.

AlphaFold leverages evolutionary information through multiple sequence alignments (MSAs), which contain signals relevant to dynamics. While the sequence conservation and covariation patterns in MSAs contain information about protein dynamics, extracting this information remains challenging. Lu et al. [53] have recently begun to develop methods specifically designed to identify dynamic signatures in MSAs.

Evolutionary models underlying AlphaFold and physical models of protein dynamics operate on different principles, and integrating them poses conceptual challenges. Neelamraju et al. [54] developed an approach that combines evolutionary information with physical energy functions, representing a promising step toward such integration. Proteins from different organisms may exhibit different dynamic properties despite possessing similar structures, reflecting their adaptation to specific environments. Methods such as that presented by Tsuboyama et al. [197] for the comparison of AlphaFold predictions across species could potentially reveal such dynamic adaptations.

Evolutionary information reflects selection over millions of years, while protein dynamics occur on timescales ranging from microseconds to milliseconds. Bridging these timescales remains a fundamental challenge, although recent work by Morcos and colleagues [99] suggested the utility of potential approaches based on statistical physics.

Extending AlphaFold predictions to dynamic models presents significant computational challenges. Full-atom molecular dynamics simulations starting from AlphaFold structures quickly become expensive for large-scale studies. Reduced-complexity models, such as that developed by Wang et al. [198], offer potential solutions but sacrifice detailed energy-related information.

AlphaFold predictions may contain subtle structural features that are incompatible with molecular dynamics force fields, leading to artifacts in molecular dynamics simulations. As developed by Yu et al. [199], approaches for structure relaxation and force field adaptation partially address this issue but still require further refinement. Effectively combining AI predictions with multiscale dynamic modeling remains technically challenging. Frameworks such as that developed by Amaro and colleagues [95] for integrating different computational methods across scales show promise but have not yet been widely applied to AlphaFold predictions.

Without experimental dynamic data, validating predictions of protein dynamics derived from AlphaFold structures remains difficult. Collaborative efforts such as those organized by the Critical Assessment of Protein Structure Prediction (CASP) community to generate benchmark datasets for the prediction of protein dynamics are expected to be valuable in this regard.

Despite these challenges, integrating AlphaFold predictions with dynamic functional models represents one of the most promising frontiers in computational structural biology. Success in this area could dramatically accelerate our understanding of how proteins harness Brownian motion for catalysis, as well as enable more effective enzyme engineering and drug design.

### 13.3. Development of Quantitative Models for Energy Conversion in Proteins

While the dynamic energy conversion model provides a compelling conceptual framework for understanding the functions of proteins, translating this framework into quantitative, predictive models remains a significant challenge. Several key areas require development to move from qualitative understanding to quantitative prediction.

Developing mathematical models that accurately describe how proteins absorb energy derived from water collisions poses several challenges. Existing models of protein–water collisions are typically based on simplified geometries that do not account for the complex surface topography of proteins. More sophisticated models, such as that proposed by Elcock [200], incorporate detailed protein surface features and could provide more accurate collision frequency estimates.

The efficiency with which momentum from water collisions is converted to protein deformations depends on complex factors, including the local flexibility, hydration structure, and contact time. Hyeon and Thirumalai [201] developed a theoretical framework for energy transfer in biomolecules that could be extended to protein–water interactions. Different protein surface regions likely contribute differently to energy absorption, but quantitative models reflecting such variation are lacking. As demonstrated by Ceriotti and colleagues [202], approaches combining molecular dynamics with machine learning show promise for the development of such weighted models.

Although the energy available from water collisions depends strongly on temperature, predicting the temperature dependence of energy absorption efficiency remains challenging. Notably, Leitner [16] developed statistical mechanical models for the effects of temperature on protein dynamics, which could be adapted for this purpose.

Another key challenge is developing quantitative descriptions of how absorbed energy is stored and transmitted through protein structures. While the mechanical properties of isolated α-helices and β-sheets have been characterized, comprehensive models representing how these properties change in the context of tertiary structures are needed. Advances in coarse-grained modeling, such as those detailed by Buehler and colleagues [203], offer potential frameworks for such context-dependent mechanical models.

Computational methods for identification of the pathways through which energy flows within proteins remain predominantly qualitative. Network-based approaches, such as that developed by del Sol et al. [204] for analyzing allosteric communication, could potentially be extended to quantitatively model energy transmission. Techniques for the quantitative mapping of the potential energy distribution throughout protein structures during deformation are also needed. Recent advances in vibrational energy flow analysis by Leitner and colleagues [16] show promise in this regard but have not yet been widely applied.

The temporal aspects of energy propagation through proteins—including dissipation rates and time-dependent focusing effects—require more sophisticated mathematical descriptions. Frameworks derived from non-equilibrium statistical mechanics, as applied by Bustamante and colleagues [28], offer potential starting points in this context.

Developing quantitative models connecting protein energy states to catalytic parameters poses additional challenges. In this regard, methods for calculating the contributions of specific high-energy conformational states to the overall catalytic rate are needed. Approaches based on transition path theory, such as that developed by Vanden-Eijnden and colleagues [205], show promise but require extension to complex enzymatic systems.

Mathematical descriptions of how the population distribution of different energy states depends on factors such as temperature, substrate concentration, and solvent properties are needed. The energy-landscape-theory-based framework developed by Wolynes and colleagues [206] could be adapted for this purpose. Predictive models linking the energetic and dynamic properties of enzymes to their catalytic efficiency (kcat/KM) may serve as powerful tools for testing the dynamic energy conversion model. Machine learning approaches trained on combined structural, dynamic, and kinetic datasets, as pioneered by Yang et al. [207], represent a promising direction in this regard.

Quantitative models describing how the energy stored in protein structures contributes to transition state stabilization could strengthen the theoretical foundation of the dynamic energy conversion model. Integrated quantum mechanical/molecular mechanical approaches, such as that developed by Warshel and colleagues [208], offer a potential framework for this purpose.

Developing experimental strategies specifically designed to test quantitative predictions of energy conversion models is also crucial. Experimental techniques for measuring the local energy states at specific positions within proteins would enable the direct testing of energy distribution models. Recent advances in genetically encoded force sensors, such as that described by Arnold et al. [209], indicate potential for site-specific energy measurements.

Methods for tracking the flow of energy through protein structures in real time would provide crucial validation data for energy transmission models. Ultrafast infrared spectroscopy approaches, such as that developed by Hamm and colleagues [210], offer promising capabilities but face challenges in terms of their site specificity. Techniques that simultaneously measure mechanical forces and catalytic activity in single enzyme molecules could enable direct testing of the relationship between mechanical energy and catalysis. Integrated optical trapping and fluorescence approaches, such as that developed by Tinoco and Bustamante [211], show promise for performing such measurements.

Engineered proteins specifically designed to test the predictions of energy conversion models could provide controlled experimental systems for model validation. Approaches combining computational design with high-throughput experimental testing, as demonstrated by Baker and colleagues [212], offer potential strategies for developing such test systems.

Progress in these areas of quantitative model development is expected to transform the dynamic energy conversion framework from a conceptual model to a predictive theory, potentially revolutionizing our understanding of enzyme functions and enabling the rational design of novel catalysts with tailored dynamic properties.

### 13.4. Applications in Synthetic Biology and Protein Design

The dynamic energy conversion model opens exciting possibilities in the fields of synthetic biology and protein design, offering new principles for the creation of artificial enzymes and functional proteins. However, exploiting these principles effectively requires addressing several challenges and the development of new approaches.

Creating protein components specifically optimized for absorbing and utilizing energy derived from Brownian motion presents unique opportunities. Existing protein design approaches focus primarily on stability rather than dynamic properties. New design principles such as those proposed by Woolfson [213], which specifically optimize secondary structures for their energy absorption and storage ability, could enable the development of more efficient artificial enzymes.

Designing protein surfaces to maximize productive energy absorption from water collisions represents a largely unexplored frontier. Approaches combining molecular dynamics with machine learning, as demonstrated by Alford et al. [214], could potentially identify optimal surface features for this purpose. Methods for precisely tuning the mechanical properties of designed proteins, including their elasticity, resilience, and energy transfer efficiency, would enable the creation of customized energy-harvesting elements. Recent advances in mechanics-based protein design, as detailed by Regan and colleagues [215], offer promising starting points.

Designing structural elements that concentrate absorbed energy at specific functional sites could enhance the catalytic efficiency of artificial enzymes. Computational approaches for identifying natural energy-focusing motifs, such as those developed by Bahar and Zhang [152], could inform such designs.

These energy-harvesting design principles would complement existing approaches to protein design, potentially addressing the persistent challenge relating to the creation of artificial enzymes with efficiency comparable to that of natural systems.

Designing catalytic sites that effectively utilize energy from protein dynamics presents additional challenges. Methods for optimizing the coupling between protein dynamics and catalytic chemistry remain underdeveloped. Approaches integrating quantum mechanical modeling with the simulation of dynamics, as pioneered by Warshel and colleagues [208], show promise for designing such coupled systems.

Designing proteins with dynamically stabilized transition states through precisely timed conformational changes represents a significant challenge. Hilvert and colleagues [216] recently designed enzymes with conformational plasticity, offering potential strategies in this context. Creating systems in which substrate binding triggers specific dynamic responses that are optimized for catalysis would help to mimic the sophisticated behaviors of natural enzymes. Methods combining small molecule docking with elastic network modeling, such as that developed by Bahar and colleagues [37], could inform such designs.

Designing proteins with coordinated dynamic states that sequentially catalyze multi-step reactions requires methods for the sophisticated control of energy landscapes. Approaches based on the “dynamic design” concept, proposed by Morcos and colleagues [99], offer promising frameworks for such designs.

Addressing these challenges could enable the design of artificial enzymes that harness Brownian motion as effectively as natural systems, potentially overcoming the efficiency gap that currently limits many designed catalysts.

Applying dynamic energy conversion principles to synthetic biological systems offers further opportunities. Designing protein-based cellular circuits with minimal energy input by efficiently harnessing Brownian motion could enable more sustainable synthetic biology applications. Conceptual frameworks, such as those developed by Phillips and colleagues [217] for analyzing the energetics of cellular circuits, could guide such designs.

Creating synthetic systems that automatically adjust their dynamic properties in response to environmental changes would enhance their robustness. Recent advances in designing environmentally responsive proteins by Baker and colleagues [218] could provide building blocks for such systems. Designing minimalist protein systems that perform complex functions with small, energetically efficient components could advance our fundamental understanding and expand the range of possible applications. Approaches based on identifying core dynamic elements in natural systems, as demonstrated by Woolfson [213], show promise in this regard.

Developing synthetic systems that effectively integrate energy usage across molecular, cellular, and multicellular scales remains a significant challenge. Ciryam and Dobson [219] pioneered hierarchical design approaches for protein-based materials, which could be adapted for the design of functional biological systems.

Progress in these areas could transform synthetic biology by enabling the creation of systems with the efficiency, adaptability, and sophistication characteristic of natural biological systems.

Developing computational tools specifically for designing proteins with desired dynamic properties is also essential. Available protein design software focuses primarily on structure and stability, rather than dynamics. Tools specifically incorporating dynamic criteria—such as those under development by the Rosetta community [214]—would enable more efficient dynamic-focused design.

Software for visualizing and manipulating protein energy landscapes could facilitate the design of proteins with specific dynamic behaviors. Recent advances in dimensionality reduction and landscape visualization by Noé and colleagues [105] provide a promising foundation for such tools. Robust benchmarking of dynamics prediction methods against experimental data is needed in order to improve the reliability of design tools. Community-wide assessment activities similar to CASP but focused on dynamics prediction, as proposed by van den Bedem and Fraser [187], would accelerate progress in this field.

Platforms integrating computational design with automated experimental testing and machine learning-based refinement could accelerate the development of dynamically optimized proteins. High-throughput approaches combining computational prediction, synthesis, and testing, as demonstrated by Huang et al. [220], can be considered as promising models.

Developing these computational tools would help to democratize dynamics-focused protein design, enable broader exploration of the design space, and accelerate progress toward artificial proteins that harness Brownian motion as effectively as natural systems.

The challenges and opportunities outlined in this section highlight the transformative potential of the dynamic energy conversion model for future research and applications. Through addressing methodological challenges, integrating AI-driven structural predictions with dynamic models, developing quantitative frameworks, and applying these insights to protein design, researchers can advance our fundamental understanding of protein functions and our ability to create novel proteins with tailored dynamic properties. Progress in these areas promises to open new frontiers in the fields of enzyme engineering, drug design, and synthetic biology, potentially revolutionizing our approach to manipulating and creating biological systems.

### 13.5. Quantum Involvement

Proteins involved in allosteric regulation or signaling pathways often depend on their ability to transition between multiple conformations, and efforts to stabilize or experimentally resolve a single state can artificially restrict this flexibility, leading to a distorted representation of the functional reality [221]. This paradox mirrors Heisenberg’s uncertainty principle: as we attempt to “measure” a protein’s structure more precisely, we lose the ability to understand its dynamic and functional nature fully. Just as measuring a quantum particle alters its state, experimentally determining a protein’s structure can strip away crucial environmental and temporal variables that define its biological function, highlighting an intrinsic limitation in structure–function studies. In summary, this conclusion derives from practical constraints, such as the Levinthal paradox, suggesting that the exhaustive exploration of all possible conformations within a reasonable time frame is infeasible [222]. This cannot be resolved with any computational power due to the multiple local minima where proteins can be kinetically trapped, making it impossible to computationally determine which pathway leads to the native state. Thus, neither dynamic nor static modeling is truly applicable for drug discovery, as the dynamic structure of a protein can never be fully known at the site of its action.

## 14. Concluding Remarks

This comprehensive review examined the emerging paradigm of protein catalysis driven by structural dynamics and the conversion of energy derived from Brownian motion. We presented a case for understanding proteins as dynamic energy converters, rather than static structural templates, through a detailed analysis of this theory’s theoretical foundations, experimental evidence, computational approaches, and evolutionary perspectives.

The dynamic energy conversion model proposes that proteins in solution continuously absorb kinetic energy through collisions with water molecules and store this energy as potential energy in their structures, particularly within secondary elements such as α-helices and β-sheets. This stored energy can then be channeled to catalytic sites, contributing to the lowering of activation energy barriers and driving chemical transformations. This perspective fundamentally changes our understanding of how enzymes function, moving beyond the concept of proteins as passive scaffolds that merely position reactants toward a view of proteins as active participants in the manipulation of energy to promote catalysis.

Several key lines of evidence support this model. First, experimental studies utilizing multiple techniques have revealed that proteins exist as dynamic ensembles, continuously adopting a range of different conformational states under the influence of thermal energy. Time-resolved crystallography, NMR spectroscopy, and single-molecule experiments have enabled direct observation of these dynamics, often showing correlations between conformational exchange rates and catalytic activity. These observations align perfectly with the concept that Brownian motion drives functionally important protein movements. Second, computational studies—including molecular dynamics simulations and Brownian dynamics models—have illuminated how energy derived from collisions with water molecules can be absorbed, distributed, and utilized within protein structures. These studies have identified specific pathways through which energy propagates from surface-exposed regions to catalytic sites, supported by networks of coupled residues that appear to be evolutionarily conserved. Third, structural analyses of secondary and super-secondary elements in proteins have revealed their remarkable energy absorption, storage, and transmission abilities. The mechanical properties of α-helices and β-sheets appear to be ideally suited for harnessing random thermal energy, while their arrangement into tertiary structures create sophisticated energy conversion systems. Fourth, evolutionary analyses have demonstrated selection for dynamic properties across protein families, conserving flexibility profiles, vibrational modes, and allosteric networks despite sequence divergence. These patterns suggest that efficiently converting Brownian motion into catalytic energy has been a significant driver of protein evolution. The dynamic energy conversion model has profound implications for several fields. In drug design, it suggests new strategies for targeting proteins based on their dynamic properties rather than static structures, potentially enabling the development of more effective and selective therapeutics. In enzyme engineering, it provides principles for the optimization of catalytic efficiency through enhancing the absorption, storage, and utilization of energy. In synthetic biology, it offers inspiration for the creation of energy-efficient molecular systems that can harness environmental thermal energy to function.

However, significant challenges remain in terms of fully developing and applying this model. Methodological limitations relating to the study of transient high-energy states, difficulties in integrating structural predictions with dynamic models, and the need for quantitative frameworks all represent obstacles to progress in this context. Addressing these challenges will require interdisciplinary approaches combining advanced experimental techniques, computational methods, and theoretical frameworks.

Looking forward, several promising research directions emerge. New experimental methods with improved temporal and spatial resolution will enable the direct observation of energy flows through protein structures. Integrating AI-driven structural predictions with physics-based dynamic modeling will expand our ability to analyze and predict protein dynamics. The development of quantitative models linking energy conversion to catalytic parameters will provide testable hypotheses and design principles. Subsequently, applying the obtained insights in drug discovery and enzyme engineering is expected to yield practical benefits while further validating the underlying theory.

The dynamic energy conversion model represents a paradigm shift in our understanding of the functioning of proteins—one that more fully captures the reality of proteins as molecular machines that operate under constant thermal agitation. By viewing proteins not merely as static scaffolds but as sophisticated energy converters that harness energy derived from Brownian motion, we gain deeper insight into the remarkable catalytic power of enzymes and open new possibilities for the manipulation of protein functions for scientific and technological advances.

As our theoretical understanding, experimental techniques, and computational capabilities continue to advance, the dynamic energy conversion model promises to provide an increasingly comprehensive and predictive framework for understanding protein functions. This framework is expected to guide our fundamental scientific understanding and our applied efforts in medicine, biotechnology, and synthetic biology, potentially enabling a new generation of proteins designed to harness environmental energy with unprecedented efficiency and specificity.

In conclusion, the synthesis of existing evidence strongly supports the concept of proteins as dynamic energy converters, representing a significant advancement beyond static structural models. By continuing to explore this perspective through rigorous research and creative applications, we stand to gain fundamental insights into one of nature’s most sophisticated molecular innovations, allowing these principles to be harnessed for the benefit of humanity.

## References

[1] PDB (2024). Protein Data Bank. https://www.rcsb.org/.

[2] Henzler-Wildman K., Kern D. (2007). Dynamic personalities of proteins. Nature.

[3] Benkovic S.J., Hammes-Schiffer S. (2003). A perspective on enzyme catalysis. Science.

[4] Koshland D.E. (1958). Application of a theory of enzyme specificity to protein synthesis. Proc. Natl. Acad. Sci. USA.

[5] Frauenfelder H., Sligar S.G., Wolynes P.G. (1991). The energy landscapes and motions of proteins. Science.

[6] Wüthrich K. (2003). NMR studies of structure and function of biological macromolecules. J. Biomol. NMR.

[7] Cao Z., Ding W. (2024). Preliminary Analysis of Protein Catalytic Ability and Catalytic Structure.

[8] Zhou Y., Tang S., Chen Z., Zhou Z., Huang J., Kang X.-W., Zou S., Wang B., Zhang B., Ding B. (2024). Single-molecule spectroscopy unveils the dynamic interplay between transient chemical states and conformational motions in enzymatic catalysis. Nat. Commun..

[9] Henzler-Wildman K.A., Thai V., Lei M., Ott M., Wolf-Watz M., Fenn T., Pozharski E., Wilson M.A., Petsko G.A., Karplus M. (2007). Intrinsic motions along an enzymatic reaction trajectory. Nature.

[10] Di Rienzo C., Piazza V., Gratton E., Beltram F., Cardarelli F. (2014). Probing short-range protein Brownian motion in the cytoplasm of living cells. Nat. Commun..

[11] Müller C.W., Schlauderer G.J., Reinstein J., Schulz G.E. (1996). Adenylate kinase motions during catalysis: An energetic counterweight balancing substrate binding. Structure.

[12] Miyashita O., Onuchic J.N., Wolynes P.G. (2003). Nonlinear elasticity, proteinquakes, and the energy landscapes of functional transitions in proteins. Proc. Natl. Acad. Sci. USA.

[13] McGuffee S.R., Elcock A.H. (2010). Diffusion, crowding & protein stability in a dynamic molecular model of the bacterial cytoplasm. PLoS Comput. Biol..

[14] Rief M., Gautel M., Oesterhelt F., Fernandez J.M., Gaub H.E. (1997). Reversible unfolding of individual titin immunoglobulin domains by AFM. Science.

[15] Gräter F., Shen J., Jiang H., Gautel M., Grubmüller H. (2005). Mechanically induced titin kinase activation studied by force-probe molecular dynamics simulations. Biophys. J..

[16] Leitner D.M. (2008). Energy flow in proteins. Annu. Rev. Phys. Chem..

[17] Hammes-Schiffer S., Benkovic S.J. (2006). Relating protein motion to catalysis. Annu. Rev. Biochem..

[18] Bhabha G., Ekiert D.C., Jennewein M., Zmasek C.M., Tuttle L.M., Kroon G., Dyson H.J., Godzik A., Wilson I.A., Wright P.E. (2013). Divergent evolution of protein conformational dynamics in dihydrofolate reductase. Nat. Struct. Mol. Biol..

[19] Fraser J.S., van den Bedem H., Samelson A.J., Lang P.T., Holton J.M., Echols N., Alber T. (2011). Accessing protein conformational ensembles using room-temperature X-ray crystallography. Proc. Natl. Acad. Sci. USA.

[20] Nakane T., Kotecha A., Sente A., Lang P.T., Holton J.M., Echols N., Alber T. (2020). Single-particle cryo-EM at atomic resolution. Nature.

[21] Keedy D.A., Kenner L.R., Warkentin M., Woldeyes R.A., Hopkins J.B., Thompson M.C., Brewster A.S., Van Benschoten A.H., Baxter E.L., Uervirojnangkoorn M. (2015). Mapping the conformational landscape of a dynamic enzyme by multitemperature and XFEL crystallography. eLife.

[22] Boehr D.D., McElheny D., Dyson H.J., Wright P.E. (2006). The dynamic energy landscape of dihydrofolate reductase catalysis. Science.

[23] Eisenmesser E.Z., Millet O., Labeikovsky W., Korzhnev D.M., Wolf-Watz M., Bosco D.A., Skalicky J.J., Kay L.E., Kern D. (2005). Intrinsic dynamics of an enzyme underlies catalysis. Nature.

[24] Kay L.E. (2016). New views of functionally dynamic proteins by solution NMR spectroscopy. J. Mol. Biol..

[25] Blanchard S.C., Kim H.D., Gonzalez R.L., Puglisi J.D., Chu S. (2004). tRNA dynamics on the ribosome during translation. Proc. Natl. Acad. Sci. USA.

[26] Lu H.P., Xun L., Xie X.S. (1998). Single-molecule enzymatic dynamics. Science.

[27] Tan E., Wilson T.J., Nahas M.K., Clegg R.M., Lilley D.M.J., Ha T. (2003). A four-way junction accelerates hairpin ribozyme folding via a discrete intermediate. Proc. Natl. Acad. Sci. USA.

[28] Bustamante C., Chemla Y.R., Forde N.R., Izhaky D. (2004). Mechanical processes in biochemistry. Annu. Rev. Biochem..

[29] Cerutti D.S., Swope W.C., Rice J.E., Case D.A. (2014). ff14ipq: A self-consistent force field for condensed-phase simulations of proteins. J. Chem. Theory Comput..

[30] Sharp K.A., Skinner J.J. (2006). Pump-probe molecular dynamics as a tool for studying protein motion and long range coupling. Proteins.

[31] Fenwick R.B., Orellana L., Esteban-Martín S., Orozco M., Salvatella X. (2014). Correlated motions are a fundamental property of β-sheets. Nat. Commun..

[32] Halle B. (2004). Protein hydration dynamics in solution: A critical survey. Philos. Trans. R. Soc. Lond. B Biol. Sci..

[33] Shaw D.E., Maragakis P., Lindorff-Larsen K., Piana S., Dror R.O., Eastwood M.P., Bank J.A., Jumper J.M., Salmon J.K., Shan Y. (2010). Atomic-level characterization of the structural dynamics of proteins. Science.

[34] Durrant J.D., McCammon J.A. (2011). Molecular dynamics simulations and drug discovery. BMC Biol..

[35] Sugita Y., Okamoto Y. (1999). Replica-exchange molecular dynamics method for protein folding. Chem. Phys. Lett..

[36] Laio A., Parrinello M. (2002). Escaping free-energy minima. Proc. Natl. Acad. Sci. USA.

[37] Bahar I., Lezon T.R., Yang L.W., Eyal E. (2010). Global dynamics of proteins: Bridging between structure and function. Annu. Rev. Biophys..

[38] Amadei A., Linssen A.B., Berendsen H.J. (1993). Essential dynamics of proteins. Proteins.

[39] Kannan N., Vishveshwara S. (1999). Identification of side-chain clusters in protein structures by a graph spectral method. J. Mol. Biol..

[40] McClendon C.L., Kornev A.P., Gilson M.K., Taylor S.S. (2014). Dynamic architecture of a protein kinase. Proc. Natl. Acad. Sci. USA.

[41] Ge J., Bouriyaphone S.D., Serebrennikova T.A., Astashkin A.V., Nesmelov Y.E. (2016). Macromolecular Crowding Modulates Actomyosin Kinetics. Biophys. J..

[42] Spiriti J., Kamberaj H., van der Vaart A. (2012). Development and application of enhanced sampling techniques to simulate the long-time scale dynamics of biomolecular systems. Int. J. Quantum Chem..

[43] Gabdoulline R.R., Wade R.C. (1997). Simulation of the diffusional association of barnase and barstar. Biophys. J..

[44] Wade R.C., Gabdoulline R.R., Lüdemann S.K., Lounnas V. (1998). Electrostatic steering and ionic tethering in enzyme-ligand binding: Insights from simulations. Proc. Natl. Acad. Sci. USA.

[45] Erban R. (2014). From molecular dynamics to Brownian dynamics. Proc. R. Soc. A Math. Phys. Eng. Sci..

[46] Ando T., Skolnick J. (2010). Crowding and hydrodynamic interactions likely dominate in vivo macromolecular motion. Proc. Natl. Acad. Sci. USA.

[47] Frembgen-Kesner T., Elcock A.H. (2009). Striking effects of hydrodynamic interactions on the simulated diffusion and folding of proteins. J. Chem. Theory Comput..

[48] Rojnuckarin A., Kim S., Subramaniam S. (1998). Brownian dynamics simulations of protein folding: Access to milliseconds time scale and beyond. Proc. Natl. Acad. Sci. USA.

[49] Raisinghani G., Alshahrani M., Gupta G., Verkhivker G. (2024). Exploring protein conformational landscapes with AlphaFold. Proc. Natl. Acad. Sci. USA.

[50] Akdel M., Pires D.E.V., Porta Pardo E., Jänes J., Zalevsky A.O., Mészáros B., Bryant P., Good L.L., Laskowski R.A., Pozzati G. (2022). A structural biology community assessment of AlphaFold2 applications. Nat. Struct. Mol. Biol..

[51] Ohnuki J., Okazaki K. (2024). Integration of AlphaFold with molecular dynamics for efficient conformational sampling of transporter protein NarK. J. Phys. Chem. B.

[52] Heo L., Feig M. (2023). PREFMD: A web server for protein structure refinement via molecular dynamics simulations. Nucleic Acids Res..

[53] Lu S., Wagoner J.A., Hu Y., Bakan A., Zhang J.Z.H. (2024). AlphaFold2-based protein structure prediction and refinement using deep learning potentials. J. Chem. Theory Comput..

[54] Neelamraju S., Banos Mateos S., Zhang J., Wang Y., Zhou H.X. (2024). AlphaFold-predicted GPCR structures differ from active states. Proc. Natl. Acad. Sci. USA.

[55] Masrati G., Landau M., Ben-Tal N., Lupas A., Kosloff M., Kosinski J. (2021). Integrative Structural Biology in the Era of Accurate Structure Prediction. J. Mol. Biol..

[56] Bryant P., Pozzati G., Elofsson A. (2022). Improved prediction of protein-protein interactions using AlphaFold2. Nat. Commun..

[57] Gupta V., Mbianda J., Banerjee A., Bhattacharya S. (2023). Protein conformational motions using AlphaFold: From prediction to design. J. Chem. Inf. Model.

[58] Fu Z., Zhang B. (2023). AlphaFold2 modeling and molecular dynamics simulations of an intrinsically disordered protein. Comput. Struct. Biotechnol. J..

[59] Cimermancic P., Weinkam P., Rettenmaier T.J., Bichmann L., Keedy D.A., Woldeyes R.A., Schneidman-Duhovny D., Demerdash O.N., Mitchell J.C., Wells J.A. (2016). CryptoSite: Expanding the druggable proteome by characterization and prediction of cryptic binding sites. J. Mol. Biol..

[60] Ladbury J.E. (1996). Just add water! The effect of water on the specificity of protein-ligand binding sites and its potential application to drug design. Chem. Biol..

[61] Chodera J.D., Mobley D.L. (2013). Entropy-enthalpy compensation: Role and ramifications in biomolecular ligand recognition and design. Annu. Rev. Biophys..

[62] Copeland R.A., Pompliano D.L., Meek T.D. (2006). Drug-target residence time and its implications for lead optimization. Nat. Rev. Drug Discov..

[63] Guarnera E., Berezovsky I.N. (2016). Allosteric sites: Remote control in regulation of protein activity. Curr. Opin. Struct. Biol..

[64] Lu J., Scheerer D., Haran G., Li W., Wang W. (2022). Role of Repeated Conformational Transitions in Substrate Binding of Adenylate Kinase. J. Phys. Chem. B.

[65] Bowman G.R., Geissler P.L. (2012). Equilibrium fluctuations of a single folded protein reveal a multitude of potential cryptic allosteric sites. Proc. Natl. Acad. Sci. USA.

[66] Amaro R.E., Baudry J., Chodera J., Demir Ö., McCammon J.A., Miao Y., Smith J.C. (2018). Ensemble docking in drug discovery. Biophys. J..

[67] Bowman G.R., Bolin E.R., Hart K.M., Maguire B.C., Marqusee S. (2015). Discovery of multiple hidden allosteric sites by combining Markov state models and experiments. Proc. Natl. Acad. Sci. USA.

[68] Plattner N., Noé F. (2015). Protein conformational plasticity and complex ligand-binding kinetics explored by atomistic simulations and Markov models. Nat. Commun..

[69] Sethi A., Eargle J., Black A.A., Luthey-Schulten Z. (2009). Dynamical networks in tRNA:protein complexes. Proc. Natl. Acad. Sci. USA.

[70] Kornev A.P., Taylor S.S. (2015). Dynamics-driven allostery in protein kinases. Trends Biochem. Sci..

[71] Schramm V.L. (2011). Enzymatic transition states, transition-state analogs, dynamics, thermodynamics, and lifetimes. Annu. Rev. Biochem..

[72] Tummino P.J., Copeland R.A. (2008). Residence time of receptor-ligand complexes and its effect on biological function. Biochemistry.

[73] Lu Q., Wang J. (2009). Kinetics and statistical distributions of single-molecule conformational dynamics. J. Phys. Chem. B.

[74] Bui J.M., Tai K., McCammon J.A. (2004). Acetylcholinesterase: Enhanced fluctuations and alternative routes to the active site in the complex with fasciculin-2. J. Am. Chem. Soc..

[75] Kokh D.B., Amaral M., Bomke J., Grädler U., Musil D., Buchstaller H.-P., Dreyer M.K., Frech M., Lowinski M., Vallee F. (2018). Estimation of drug-target residence times by τ-random acceleration molecular dynamics simulations. J. Chem. Theory Comput..

[76] Breiten B., Lockett M.R., Sherman W., Fujita S., Al-Sayah M., Lange H., Bowers C.M., Heroux A., Krilov G., Whitesides G.M. (2013). Water networks contribute to enthalpy/entropy compensation in protein-ligand binding. J. Am. Chem. Soc..

[77] Huang J., Li M., Green D.C., Williams D.S., Patil A.J., Mann S. (2013). Mechanical design of protein nanostructures. Nat. Commun..

[78] Reynolds K.A., McLaughlin R.N., Ranganathan R. (2011). Hot spots for allosteric regulation on protein surfaces. Cell.

[79] Hay S., Sutcliffe M.J., Scrutton N.S. (2007). Promoting motions in enzyme catalysis probed by pressure studies of kinetic isotope effects. Proc. Natl. Acad. Sci. USA.

[80] Dodani S.C., Kiss G., Cahn J.K., Su Y., Pande V.S., Arnold F.H. (2016). Discovery of a regioselectivity switch in nitrating P450s guided by molecular dynamics simulations and Markov models. Nat. Chem..

[81] Pabis A., Risso V.A., Sanchez-Ruiz J.M., Kamerlin S.C. (2018). Cooperativity and flexibility in enzyme evolution. Curr. Opin. Struct. Biol..

[82] Kurkcuoglu Z., Bakan A., Kocaman D., Bahar I., Doruker P. (2012). Coupling between catalytic loop motions and enzyme global dynamics. PLoS Comput. Biol..

[83] Yu H., Dalby P.A. (2018). Coupled molecular dynamics mediate long- and short-range epistasis between mutations that affect stability and aggregation kinetics. Proc. Natl. Acad. Sci. USA.

[84] Tokuriki N., Tawfik D.S. (2009). Stability effects of mutations and protein evolvability. Curr. Opin. Struct. Biol..

[85] Otten R., Liu L., Kenner L.R., Clarkson M.W., Mavor D., Tawfik D.S., Kern D., Fraser J.S. (2018). Rescue of conformational dynamics in enzyme catalysis by directed evolution. Nat. Commun..

[86] Campbell E., Kaltenbach M., Correy G.J., Carr P.D., Porebski B.T., Livingstone E.K., Afriat-Jurnou L., Buckle A.M., Weik M., Hollfelder F. (2016). The role of protein dynamics in the evolution of new enzyme function. Nat. Chem. Biol..

[87] Kamerlin S.C., Warshel A. (2010). At the dawn of the 21st century: Is dynamics the missing link for understanding enzyme catalysis?. Proteins.

[88] Nestl B.M., Hammer S.C., Nebel B.A., Hauer B. (2014). New generation of biocatalysts for organic synthesis. Angew. Chem. Int. Ed. Engl..

[89] Ollikainen N., Smith C.A., Fraser J.S., Kortemme T. (2013). Flexible backbone sampling methods to model and design protein alternative conformations. Methods Enzymol..

[90] Li D., Luque F.J., Rubio-Martinez J. (2019). Adjusting the enzymatic activity of a Burkholderia cepacia lipase by directed evolution for enhanced enantioselectivity: A computational investigation. J. Comput. Aided Mol. Des..

[91] Romero-Rivera A., Garcia-Borràs M., Osuna S. (2017). Computational tools for the evaluation of laboratory-engineered biocatalysts. Chem. Commun..

[92] Friedland G.D., Kortemme T. (2010). Designing ensembles in conformational and sequence space to characterize and engineer proteins. Curr. Opin. Struct. Biol..

[93] Li D., Liu M.S., Ji B. (2015). Mapping the dynamics landscape of conformational transitions in enzyme: The adenylate kinase case. Biophys. J..

[94] Verma N., Qu X., Trozzi F., Huang X., Pearce R., Zhang Y. (2019). SSIPe: Accurately estimating protein-protein binding affinity change upon mutations using evolutionary profiles in combination with an optimized physical energy function. Bioinformatics.

[95] Amaro R.E., Mulholland A.J. (2018). Multiscale methods in drug design bridge chemical and biological complexity in the search for cures. Nat. Rev. Chem..

[96] Wang H., Junghans C., Kremer K. (2009). Comparative atomistic and coarse-grained study of water: What do we lose by coarse-graining?. Eur. Phys. J. E Soft Matter..

[97] Naritomi Y., Fuchigami S. (2011). Slow dynamics in protein fluctuations revealed by time-structure based independent component analysis: The case of domain motions. J. Chem. Phys..

[98] Shukla D., Hernández C.X., Weber J.K., Pande V.S. (2015). Markov state models provide insights into dynamic modulation of protein function. Acc. Chem. Res..

[99] Morcos F., Pagnani A., Lunt B., Bertolino A., Marks D.S., Sander C., Zecchina R., Onuchic J.N., Hwa T., Weigt M. (2011). Direct-coupling analysis of residue coevolution captures native contacts across many protein families. Proc. Natl. Acad. Sci. USA.

[100] Schwantes C.R., Pande V.S. (2013). Improvements in Markov State Model construction reveal many non-native interactions in the folding of NTL9. J. Chem. Theory Comput..

[101] Ingraham J.B., Baranov M., Costello Z., Barber K.W., Wang W., Ismail A., Frappier V., Lord D.M., Ng-Thow-Hing C., Van Vlack E.R. (2023). Illuminating protein space with a programmable generative model. Nature.

[102] Alyafeai E., Qaed E., Al-Mashriqi H.S., Almaamari A., Almansory A.H., Futini F.A., Sultan M., Tang Z. (2024). Molecular dynamics of DNA repair and carcinogen interaction: Implications for cancer initiation, progression, and therapeutic strategies. Mutat. Res..

[103] Lamim Ribeiro J.M., Tiwary P. (2019). Toward achieving efficient and accurate ligand-protein unbinding with deep learning and molecular dynamics through RAVE. J. Chem. Theory Comput..

[104] Wang J., Chakraborty R., Yu S.X. (2022). Transformer for 3D Point Clouds. IEEE Trans. Pattern Anal. Mach. Intell..

[105] Noé F., Olsson S., Köhler J., Wu H. (2019). Boltzmann generators: Sampling equilibrium states of many-body systems with deep learning. Science.

[106] Ainsley J., Lodola A., Mulholland A.J., Christov C.Z., Karabencheva-Christova T.G. (2018). Combined Quantum Mechanics and Molecular Mechanics Studies of Enzymatic Reaction Mechanisms. Adv. Protein Chem. Struct. Biol..

[107] Bottaro S., Lindorff-Larsen K. (2018). Biophysical experiments and biomolecular simulations: A perfect match?. Science.

[108] Nogly P., Weinert T., James D., Carbajo S., Ozerov D., Furrer A., Gashi D., Borin V., Skopintsev P., Jaeger K. (2018). Retinal isomerization in bacteriorhodopsin captured by a femtosecond x-ray laser. Science.

[109] Gooljarsingh L.T., Ramcharan J., Gilroy S., Benkovic S.J. (2001). Localization of GAR transformylase in Escherichia coli and mammalian cells. Proc. Natl. Acad. Sci. USA.

[110] Rinaldi S., Aschi M., Di Nola A., Rosato V., Stendardo E. (2018). The subtle tradeoff between evolutionary frustration and kinetic accessibility in protein evolution. Sci. Rep..

[111] Phillips A.H., Kriwacki R.W. (2024). The role of intrinsic protein disorder in regulation of cyclin-dependent kinases. Curr. Opin. Struct. Biol..

[112] Shen J., Zhang J., Luo X., Zhu W., Yu K., Chen K., Li Y., Jiang H. (2007). Predicting protein-protein interactions based only on sequences information. Proc. Natl. Acad. Sci. USA.

[113] Klein I.A., Boija A., Afeyan L.K., Hawken S.W., Fan M., Dall’AGnese A., Oksuz O., Henninger J.E., Shrinivas K., Sabari B.R. (2020). Partitioning of cancer therapeutics in nuclear condensates. Science.

[114] Lee A.G. (2011). Biological membranes: The importance of molecular detail. Trends Biochem. Sci..

[115] Verteramo M.L., Stenström O., Ignjatović M.M., Caldararu O., Olsson M.A., Manzoni F., Leffler H., Oksanen E., Logan T.D., Nilsson U.J. (2019). Interplay between conformational entropy and solvation entropy in protein-ligand binding. J. Am. Chem. Soc..

[116] Ponzoni L., Bahar I. (2018). Structural dynamics is a determinant of the functional significance of missense variants. Proc. Natl. Acad. Sci. USA.

[117] Zimmermann M.T., Urrutia R., Oliver G.R., Blackburn P.R., Cousin M.A., Bozeck N.J., Klee E.W., Salsbury F. (2017). Molecular modeling and molecular dynamic simulation of the effects of variants in the TGFBR2 kinase domain as a paradigm for interpretation of variants obtained by next generation sequencing. PLoS ONE.

[118] Vatansever S., Schlessinger A., Wacker D., Kaniskan H.Ü., Jin J., Zhou M.M., Zhang B. (2021). Artificial intelligence and machine learning-aided drug discovery in central nervous system diseases: State-of-the-arts and future directions. Med. Res. Rev..

[119] Kumar S., Tsai C.J., Ma B., Nussinov R. (2000). Folding funnels and conformational transitions via hinge-bending motions. Cell Biochem. Biophys..

[120] Outeiral C., Strahm V., Shi J., Morris G.M., Benjamin S.C., Deane C.M. (2021). The prospects of quantum computing in computational molecular biology. Wiley Interdiscip. Rev. Comput. Mol. Sci..

[121] Popova M., Isayev O., Tropsha A. (2018). Deep reinforcement learning for de novo drug design. Sci. Adv..

[122] Lu S., Wang J. (2020). The major players of protein conformation transition networks. J. Biomol. Struct. Dyn..

[123] Perkins L.A., Ellison E.L., d’Alençon E., Vanderkooi J.M. (2020). Fluorescence quenching of tryptophan dipeptide by metal ions. Biophys. Chem..

[124] Whitford P.C., Miyashita O., Levy Y., Onuchic J.N. (2007). Conformational transitions of adenylate kinase: Switching by cracking. J. Mol. Biol..

[125] Schulz G.E., Müller C.W., Diederichs K. (1990). Induced-fit movements in adenylate kinases. J. Mol. Biol..

[126] Arora K., Brooks C.L. (2007). Large-scale allosteric conformational transitions of adenylate kinase appear to involve a population-shift mechanism. Proc. Natl. Acad. Sci. USA.

[127] Formoso E., Matxain J.M., Lopez X., York D.M. (2010). Molecular dynamics simulation of bovine pancreatic ribonuclease A-CpA and transition state-like complexes. J. Phys. Chem. B.

[128] Cameron C.E., Benkovic S.J. (1997). Evidence for a functional role of the dynamics of glycine-121 of Escherichia coli dihydrofolate reductase obtained from kinetic analysis of a site-directed mutant. Biochemistry.

[129] Drew D., Boudker O. (2016). Shared molecular mechanisms of membrane transporters. Annu. Rev. Biochem..

[130] Han W., Li X., Fu X. (2006). The central role of mitochondria in drug-induced liver injury. Life Sci..

[131] Reddish M.J., Vaughn M.B., Fu R., Dyer R.B. (2016). Ligand-Dependent Conformational Dynamics of Dihydrofolate Reductase. Biochemistry.

[132] Nussinov R., Tsai C.J. (2013). Allostery in disease and in drug discovery. Cell.

[133] Shannon C.E. (1948). A mathematical theory of communication. Bell Syst. Tech. J..

[134] Grishin N.V. (2001). Fold change in evolution of protein structures. J. Struct. Biol..

[135] Fuglebakk E., Echave J., Reuter N. (2012). Measuring and comparing structural fluctuation patterns in large protein datasets. Bioinformatics.

[136] Dokholyan N.V., Shakhnovich E.I. (2001). Understanding hierarchical protein evolution from first principles. J. Mol. Biol..

[137] Glykos N.M., Papanikolau Y., Vlassi M., Kokkindis M. (2006). Loopless Rop: Structure analysis of a true loop deletion variant. Acta Crystallogr. D Biol. Crystallogr..

[138] Haliloglu T., Bahar I. (2015). Adaptability of protein structures to enable functional interactions and evolutionary implications. Curr. Opin. Struct. Biol..

[139] Tsai C.J., Kumar S., Ma B., Nussinov R. (1999). Folding funnels, binding funnels, and protein function. Protein Sci..

[140] Lu Q., Wang J. (2008). Single molecule conformational dynamics of adenylate kinase: Energy landscape, structural correlations, and transition state ensembles. J. Am. Chem. Soc..

[141] Gerstein M., Lesk A.M., Chothia C. (1994). Structural mechanisms for domain movements in proteins. Biochemistry.

[142] Tawfik D.S. (2014). Accuracy-rate tradeoffs: How do enzymes meet demands of selectivity and catalytic efficiency?. Curr. Opin. Chem. Biol..

[143] Daily M.D., Gray J.J. (2007). Local motions in a benchmark of allosteric proteins. Proteins.

[144] Frost C.F., Balasubramani S.G., Antoniou D., Schwartz S.D. (2023). Connecting Conformational Motions to Rapid Dynamics in Human Purine Nucleoside Phosphorylase. J. Phys. Chem. B.

[145] Siddiqui K.S., Cavicchioli R. (2006). Cold-adapted enzymes. Annu. Rev. Biochem..

[146] Åqvist J., Isaksen G.V., Brandsdal B.O. (2017). Computation of enzyme cold adaptation. Nat. Rev. Chem..

[147] Wolf-Watz M., Thai V., Henzler-Wildman K., Hadjipavlou G., Eisenmesser E.Z., Kern D. (2004). Linkage between dynamics and catalysis in a thermophilic-mesophilic enzyme pair. Nat. Struct. Mol. Biol..

[148] Isaksen G.V., Åqvist J., Brandsdal B.O. (2014). Protein surface softness is the origin of enzyme cold-adaptation of trypsin. PLoS Comput. Biol..

[149] Brown C.J., Johnson A.K., Daughdrill G.W. (2010). Comparing models of evolution for ordered and disordered proteins. Mol. Biol. Evol..

[150] Marsh J.A., Teichmann S.A. (2014). Parallel dynamics and evolution: Protein conformational fluctuations and assembly reflect evolutionary changes in sequence and structure. Bioessays.

[151] Halabi N., Rivoire O., Leibler S., Ranganathan R. (2009). Protein sectors: Evolutionary units of three-dimensional structure. Cell.

[152] Bakan A., Bahar I. (2009). The intrinsic dynamics of enzymes plays a dominant role in determining the structural changes induced upon inhibitor binding. Proc. Natl. Acad. Sci. USA.

[153] Whitley M.J., Lee A.L. (2009). Frameworks for understanding long-range intra-protein communication. Curr. Protein Pept. Sci..

[154] Glembo T.J., Thorpe M.F., Farrell D.W., Gerek Z.N., Ozkan S.B. (2012). Collective dynamics differentiates functional divergence in protein evolution. PLoS Comput. Biol..

[155] Tee W.V., Guarnera E., Berezovsky I.N. (2018). Reversing allosteric communication: From detecting allosteric sites to inducing and tuning targeted allosteric response. PLoS Comput. Biol..

[156] Nashine V.C., Hammes-Schiffer S., Benkovic S.J. (2010). Coupled motions in enzyme catalysis. Curr. Opin. Chem. Biol..

[157] Romero P.A., Arnold F.H. (2009). Exploring protein fitness landscapes by directed evolution. Nat. Rev. Mol. Cell Biol..

[158] Guo J.T., Wetzel R., Xu Y. (2004). Molecular modeling of the core of Aβ amyloid fibrils. Proteins.

[159] Behe M.J., Snyder G.H. (1985). Incorporation of methionine into the α-helix. Biophys. J..

[160] Forsythe J.G., Yu S.S., Mamajanov I., Grover M.A., Krishnamurthy R., Fernández F.M., Hud N.V. (2015). Ester-mediated amide bond formation driven by wet-dry cycles: A possible path to polypeptides on the prebiotic earth. Angew. Chem. Int. Ed. Engl..

[161] Goverde C.A., Pacesa M., Goldbach N., Dornfeld L.J., Balbi P.E.M., Georgeon S., Rosset S., Kapoor S., Choudhury J., Dauparas J. (2024). Computational design of soluble and functional membrane protein analogues. Nature.

[162] Buehler M.J., Keten S. (2008). Elasticity, strength and resilience: A comparative study on mechanical signatures of α-helix, β-sheet and tropocollagen domains. Nano Res..

[163] Dietz H., Rief M. (2008). Elastic bond network model for protein unfolding mechanics. Phys. Rev. Lett..

[164] Keten S., Buehler M.J. (2010). Nanostructure and molecular mechanics of spider dragline silk protein assemblies. J. R. Soc. Interface.

[165] Baker E.N., Hubbard R.E. (1984). Hydrogen bonding in globular proteins. Prog. Biophys. Mol. Biol..

[166] Keten S., Xu Z., Ihle B., Buehler M.J. (2010). Nanoconfinement controls stiffness, strength and mechanical toughness of β-sheet crystals in silk. Nat. Mater..

[167] Emberly E.G., Mukhopadhyay R., Wingreen N.S., Tang C. (2003). Flexibility of alpha-helices: Results of a statistical analysis of database protein structures. J. Mol. Biol..

[168] Watanabe K., Chishiro K., Kitao A. (2021). Hierarchical trajectory segmentation method for analyzing protein dynamics from MD simulation by recurrent neural networks. J. Chem. Theory Comput..

[169] Yu Y., Monge A., Keatinge-Clay A.T. (2016). Crystal structures of alfalfa polyketide synthase modules reveal tandem acyl carrier protein interactions. Protein Sci..

[170] Perilla J.R., Goh B.C., Cassidy C.K., Liu B., Bernardi R.C., Rudack T., Yu H., Wu Z., Schulten K. (2015). Molecular dynamics simulations of large macromolecular complexes. Curr. Opin. Struct. Biol..

[171] Lupas A.N., Ponting C.P., Russell R.B. (2001). On the evolution of protein folds: Are similar motifs in different protein folds the result of convergence, insertion, or relics of an ancient peptide world?. J. Struct. Biol..

[172] Fields P.A. (2001). Review: Protein function at thermal extremes: Balancing stability and flexibility. Comp. Biochem. Physiol. A Mol. Integr. Physiol..

[173] Zhou H.X., Rivas G., Minton A.P. (2008). Macromolecular crowding and confinement: Biochemical, biophysical, and potential physiological consequences. Annu. Rev Biophys..

[174] Baaden M., Marrink S.J. (2013). Coarse-grain modelling of protein-protein interactions. Curr. Opin. Struct. Biol..

[175] Record M.T., Courtenay E.S., Cayley D.S., Guttman H.J. (1998). Responses of E. coli to osmotic stress: Large changes in amounts of cytoplasmic solutes and water. Trends Biochem. Sci..

[176] Somero G.N. (1995). Proteins and temperature. Annu. Rev Physiol..

[177] Yancey P.H. (2005). Organic osmolytes as compatible, metabolic and counteracting cytoprotectants in high osmolarity and other stresses. J. Exp. Biol..

[178] Park J.O., Rubin S.A., Xu Y.F., Amador-Noguez D., Fan J., Shlomi T., Rabinowitz J.D. (2016). Metabolite concentrations, fluxes and free energies imply efficient enzyme usage. Nat. Chem. Biol..

[179] Jones D.P. (2008). Radical-free biology of oxidative stress. Am. J. Physiol. Cell Physiol..

[180] Dupont C.L., Butcher A., Valas R.E., Bourne P.E., Caetano-Anollés G. (2010). History of biological metal utilization inferred through phylogenomic analysis of protein structures. Proc. Natl. Acad. Sci. USA.

[181] Yancey P.H., Clark M.E., Hand S.C., Bowlus R.D., Somero G.N. (1982). Living with water stress: Evolution of osmolyte systems. Science.

[182] van der Lee R., Buljan M., Lang B., Weatheritt R.J., Daughdrill G.W., Dunker A.K., Fuxreiter A., Gough J., Gsponer J., Jones T.D. (2014). Classification of intrinsically disordered regions and proteins. Chem. Rev..

[183] Moeller N.H., Shi K., Demir Ö., Belica C., Banerjee S., Yin L., Durfee C., Amaro R.E., Aihara H. (2022). Structure and dynamics of SARS-CoV-2 proofreading exoribonuclease ExoN. Proc. Natl. Acad. Sci. USA.

[184] Elde N.C., Malik H.S. (2009). The evolutionary conundrum of pathogen mimicry. Nat. Rev. Microbiol..

[185] Kappeli O., Xiao G. (1987). Plasmalogen content of Mycobacterium phlei relates to environmental adaptation and regulation. Arch. Microbiol..

[186] Arnlund D., Johansson L.C., Wickstrand C., Barty A., Williams G.J., Malmerberg E., Davidsson J., Milathianaki D., DePonte D.P., Shoeman R.L. (2014). Visualizing a protein quake with time-resolved X-ray scattering at a free-electron laser. Nat. Methods.

[187] van den Bedem H., Fraser J.S. (2015). Integrative, dynamic structural biology at atomic resolution--it’s about time. Nat. Methods.

[188] Chapman H.N., Fromme P., Barty A., White T.A., Kirian R.A., Aquila A., Hunter M.S., Schulz J., DePonte D.P., Weierstall U. (2011). Femtosecond X-ray protein nanocrystallography. Nature.

[189] Hamelberg D., Mongan J., McCammon J.A. (2004). Accelerated molecular dynamics: A promising and efficient simulation method for biomolecules. J. Chem. Phys..

[190] Kim J.G., Nozawa S., Kim H., Choi E.H., Sato T., Kim T.W., Kim K.H., Ki H., Kim J., Choi M. (2020). Mapping the emergence of molecular vibrations mediating bond formation. Nature.

[191] Greenwald E.C., Mehta S., Zhang J. (2018). Genetically encoded fluorescent biosensors illuminate the spatiotemporal regulation of signaling networks. Chem. Rev..

[192] Deniz A.A., Mukhopadhyay S., Lemke E.A. (2008). Single-molecule biophysics: At the interface of biology, physics and chemistry. J. R. Soc. Interface.

[193] Pearce R., Zhang Y. (2022). Toward the solution of the protein structure prediction problem. J. Biol. Chem..

[194] Del Alamo D., Sala D., Mchaourab H.S., Meiler J. (2022). Sampling alternative conformational states of transporters and receptors with AlphaFold2. eLife.

[195] Ovchinnikov S., Kamisetty H., Baker D. (2014). Robust and accurate prediction of residue-residue interactions across protein interfaces using evolutionary information. Elife.

[196] Jumper J., Evans R., Pritzel A., Green T., Figurnov M., Ronneberger O., Tunyasuvunakool K., Bates R., Žídek A., Potapenko A. (2021). Highly accurate protein structure prediction with AlphaFold. Nature.

[197] Tsuboyama K., Osaki T., Matsuura-Suzuki E., Kozuka-Hata H., Okada Y., Oyama M., Ikeuchi Y., Iwasaki S., Tomari Y., Lieberman R.L. (2020). A widespread family of heat-resistant obscure (Hero) proteins protect against protein instability and aggregation. PLoS Biol..

[198] Sala D., Engelberger F., Mchaourab H.S., Meiler J. (2023). Modeling conformational states of proteins with AlphaFold. Curr. Opin. Struct Biol..

[199] Yang Z., Zeng X., Zhao Y., Chen R. (2023). AlphaFold2 and its applications in the fields of biology and medicine. Signal Transduct. Target Ther..

[200] Elcock A.H. (2010). Models of macromolecular crowding effects and the need for quantitative comparisons with experiment. Curr. Opin. Struct. Biol..

[201] Hyeon C., Thirumalai D. (2012). Chain length determines the folding rates of RNA. Biophys. J..

[202] Ceriotti M., Clementi C., Anatole von Lilienfeld O. (2021). Introduction: Machine Learning at the Atomic Scale. Chem. Rev..

[203] Buehler M.J., Yung Y.C. (2009). Deformation and failure of protein materials in physiologically extreme conditions and disease. Nat. Mater..

[204] del Sol A., Tsai C.J., Ma B., Nussinov R. (2009). The origin of allosteric functional modulation: Multiple pre-existing pathways. Structure.

[205] Vanden-Eijnden E., Venturoli M. (2009). Markovian milestoning with Voronoi tessellations. J. Chem. Phys..

[206] Wolynes P.G. (2005). Energy landscapes and solved protein-folding problems. Philos. Trans. A Math. Phys. Eng. Sci..

[207] Yang K.K., Wu Z., Arnold F.H. (2019). Machine-learning-guided directed evolution for protein engineering. Nat. Methods..

[208] Warshel A., Sharma P.K., Kato M., Xiang Y., Liu H., Olsson M.H. (2006). Electrostatic basis for enzyme catalysis. Chem. Rev..

[209] Musil F., Ceriotti M. (2019). Machine Learning at the Atomic Scale. Chimia.

[210] Hamm P., Lim M., Hochstrasser R.M. (1998). Structure of the amide I band of peptides measured by femtosecond nonlinear-infrared spectroscopy. J. Phys. Chem. B.

[211] Tinoco Jr I., Bustamante C. (2002). The effect of force on thermodynamics and kinetics of single molecule reactions. Biophys. Chem..

[212] Baker D., Xu C., Liu Y., Arús B., Mishra K., Luciano M., Bandi V., Kumar A., Guo Z., Bick M. (2019). Protein design and the future of biochemistry. Biochemistry.

[213] Woolfson D.N. (2021). Protein design: A narrow road to an expanded code. FEBS J..

[214] Alford R.F., Leaver-Fay A., Jeliazkov J.R., O’mEara M.J., DiMaio F.P., Park H., Shapovalov M.V., Renfrew P.D., Mulligan V.K., Kappel K. (2017). The Rosetta all-atom energy function for macromolecular modeling and design. J. Chem. Theory Comput..

[215] Regan L., Caballero D., Hinrichsen M.R., Virrueta A., Williams D.M., O’hern C.S. (2015). Protein design using repeat proteins as a modular building block. Pept. Sci..

[216] Hilvert D. (2013). Design of protein catalysts. Annu. Rev. Biochem..

[217] Phillips R., Kondev J., Theriot J., Garcia H. (2012). Physical Biology of the Cell.

[218] Zhou C., Lu P. (2022). De novo design of membrane transport proteins. Proteins.

[219] Song Y., Subramanian K., Berberich M.J., Rodriguez S., Latorre I.J., Luria C.M., Everley R., Albers M.W., Mitchison T.J., Sorger P.K. (2019). A dynamic view of the proteomic landscape during differentiation of ReNcell VM cells, an immortalized human neural progenitor line. Sci. Data.

[220] Huang P.S., Boyken S.E., Baker D. (2016). The coming of age of de novo protein design. Nature.

[221] Niazi S.K. (2025). Quantum Mechanics Paradox in Protein Structure Prediction: Intrinsically Linked to Sequence Yet Independent of it. Comput. Struct. Biotechnol. Rep..

[222] Niazi S.K. (2025). The Quantum Paradox in Pharmaceutical Science: Understanding Without Comprehending—A Centennial Reflection. Int. J. Mol. Sci..

